# Piezoelectric Biomaterial with Advanced Design for Tissue Infection Repair

**DOI:** 10.1002/advs.202413105

**Published:** 2025-01-31

**Authors:** Siyuan Shang, Fuyuan Zheng, Wen Tan, Zhengyi Xing, Siyu Chen, Fuli Peng, Xiang Lv, Duan Wang, Xiangdong Zhu, Jiagang Wu, Zongke Zhou, Xingdong Zhang, Xiao Yang

**Affiliations:** ^1^ National Engineering Research Center for Biomaterials Sichuan University Chengdu 610064 China; ^2^ Sports Medicine Center West China Hospital, Sichuan University Chengdu 610065 China; ^3^ Orthopedic Research Institute and Department of Orthopedics West China Hospital, Sichuan University Chengdu 610041 China; ^4^ Department of Burn and Plastic Surgery West China School of Medicine West China Hospital, Sichuan University Chengdu 610041 China; ^5^ College of Materials Science and Engineering Sichuan University Chengdu 610065 China; ^6^ College of Physics Sichuan University Chengdu 610065 China

**Keywords:** antibacterial, nanomaterial, piezocatalysis, piezoelectricity, reactive oxygen species

## Abstract

Bacterial infection has become the most dangerous factor in tissue repair, which strongly affects the tissue regeneration efficiency and wellness of patients. Piezoelectric materials exhibit the outstanding advantage of producing electrons without external power supply. The ability of electron enrichment and reactive oxygen species generation through noninvasive stimulations enables piezoelectric materials the potential applications of antibacterial. Many studies have proved the feasibility of piezoelectric materials as a functional addition in antibacterial biomaterial. In fact, numerous piezoelectric materials with ingenious designs are reported to be effective in antibacterial processes. This review summarizes the antibacterial mechanisms of piezoelectric, illuminating their potential in combating bacteria. Recent advancement in the design and construction of piezoelectric biomaterial including defect engineering, heterojunction, synergy with metal and the composite scaffold configuration are thoroughly reviewed. Moreover, the applications and therapeutic effects of piezoelectric materials in common tissues with antibacterial requirements are introduced, such as orthopedics, dental, and wound healing. Finally, the development prospects and points deserving further exploration are listed. This review is expected to provide valuable insight into the relationship between antibacterial processes and piezoelectric materials, further inspiring constructive development in this emerging scientific discipline.

## Graphical abstract

This review offers a comprehensive overview of piezoelectric materials in antibacterial treatments, emphasizing the configuration of piezoelectric biomaterial, the mechanisms behind their antibacterial effects, and their current applications in tissue engineering. It highlights the integration of piezoelectric materials into tissue engineering scaffolds as a promising strategy to enhance antibacterial responses and improve tissue healing outcomes.

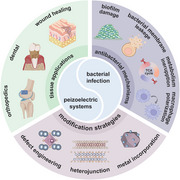

## 1 Introduction

Rapid development of tissue engineering provides a novel solution for necrotic or diseased tissue. The consequential complications of tissue infection have severely hindered the clinical efficacy of the implantation. Tissue infection always occurs in orthopedics, dentistry, and plastic surgery with high incidence, including subset classifications of peri‐prosthetic joint infection, fracture‐related infection, diabetic foot infection, periodontitis, wound infection and so on. Upon infection, a series of local immune responses rise regarding inflammation, granulation tissue formation, and fibrous encapsulation. The relevant microenvironment is conducive to bacterial settlement and lead to some detrimental consequences to both host tissue and implantation such as delayed healing, implant failure and potentially fatal systemic infection.^[^ ^1^, ^2^, ^3^ ^]^ Without effective action, the burden of deaths from antimicrobial resistance is estimated to escalate to 10 million lives each year by 2050, far exceeding the number of people dying from cancer.^[^ ^4^ ^]^ Tissue infection pose a serious threat for patients’ wellness and also results in substantial financial losses for healthcare systems. It is reported that the annual cost for implant‐associated infections (IAIs) treatment is anticipated to surpass 3.3 billion in the United States, 1.86 billion among them for orthopedic IAIs.^[^ ^5^ ^]^

Tissue infection shows connected relationships with bacteria invasion, especially *Staphylococcus aureus* (*S. aureus*), *Escherichia coli* (*E. coli*), and *Pseudomonas aeruginosa* (*P. aeruginosa*).^[^ ^6^ ^]^ Present clinical treatments for infection are restrict to antibiotics in chemical way, which are administered orally or intravenously.^[^ ^7^ ^]^ However, the colonization of bacteria on the surface of implantation will give rise to bacterial biofilms. As reported by the National Institutes of Health (CA), about 80% of microbial infections can be attributed to the existence of biofilms.^[^ ^8^ ^]^ With denser 3D network, biofilm prevents antibiotics diffusion from reaching the bacteria, and inhibit the nutritive compound consumption of cell at the same time, making it less effective for antibiotics against bacterial infections.^[^ ^9^ ^]^ This serious issue exacerbates the overuse of antibiotics, which not only threatens the body wellness due to their toxicity, but also further improves the resistance of bacteria and gives rise to multidrug‐resistant bacteria posing harm for both ecological and human health. Given the treatment of antibiotics systemic administration cannot meet the demand of preciseness and efficiency, alternative approaches are desperately needed for the effective treatment of tissue infection.

In order of infection treatment with accuracy and efficiency, many efforts have been given to the design of implant with antibacterial ability. Start from the surface modification, many antibiofouling surfaces have been developed, drawing inspiration from natural textures found in cicada wings, shark skin, gecko skin, and lotus leaves.^[^ ^10^ ^]^ These biomimic surface are mostly hydrophobic and discourages cell adhesion while bactericidal action simultaneously. Coating is another attempt. Antimicrobial peptides, nitric oxide, and quaternary amines are immobilized on the surface to inhibit bacterial attachment but show limited effect because of the difficulty in sustaining long‐term release profile. Metal ion doping like silver, aluminum, cobalt, zinc can permeate biofilm and kill bacteria, but toxic toward human organs.

Recently, the research direction evolved towards the exploration of functional nanomaterials endowed with antimicrobial properties. Materials used in catalysis and environment fields have been utilized to investigate their antibacterial function and apply to biomedical implant. Functional responsive materials are attempted to apply in antibacterial treatment and exhibited notable advantages, for example typical photosensitizers like graphene oxide and TiO_2_ nanotubes, magnetic responsive materials like Fe_3_O_4_ and so on.^[^ ^11^, ^12^, ^13^, ^14^ ^]^ They are sensitive to external stimulations in forms of light, thermal, sound, magnetic and share common antibacterial pathways of ROS generation and oxidative stress.

Piezoelectric material is one of the promising antibacterial candidates with electromechanical conversion capacity. Piezoelectricity is described as the ability of a material to transform mechanical stimulation into electrical signals, which was first discovered by the Curie brothers Pierre and Jacques in 1880.^[^ ^15^, ^16^ ^]^ A critical feature of piezoelectric material is the non‐centrosymmetric crystal structure of a unit cell. Upon a mechanical force applied, the crystal deformation occurs, then the positive and negative charges centers separate, resulting in a dipole moment.^[^ ^17^ ^]^ Piezoelectric materials have been widely applied in electronic, energy, and sensor fields so far. As it can generate endogenous electric field via nonintrusive methods rather than an external power supply,^[^ ^18^ ^]^ there is great potential for piezoelectric materials in biomedical use.^[^ ^19^ ^]^ The multifaceted biofunctions of piezoelectric implantations have been extensively demonstrated. They can serve as nanogenerators producing electric signals as rehabilitative exercises to expedite tissue healing,^[^ ^20^ ^]^ or promote in vivo osseointegration through its electropositive surfaces.^[^ ^21^ ^]^ Moreover, certain progresses have been made in the antitumor application of piezoelectric material,^[^ ^22^, ^23^ ^]^ as it shows ROS generation ability through piezoelectric catalysis, which lays a foundation for antimicrobial applications in piezoelectric materials.^[^ ^24^ ^]^ The surface charges derived from external stimulation together with the ROS generated from piezoelectric effect are both contributing to antimicrobial effect through multiple pathways. The antibacterial applications of piezoelectric materials have been designed and applied to tissues including bone, teeth and skin.

**Figure 1:**
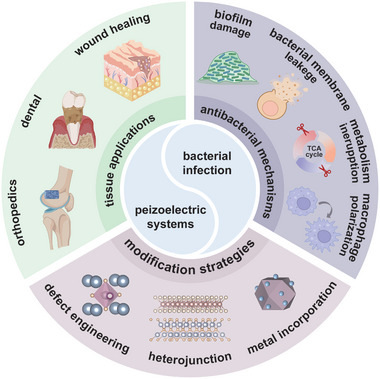
Schematic of the applications of piezoelectric biomaterial in bacterial infection, including the material design method, possible antibacterial working mechanisms and common infected tissues they target.

This review outlines the mechanism and applications of piezoelectric materials in antibacterial field (**Figure** **1**). To start with, typical piezoelectric materials with antibacterial applications and their piezoelectricity stimulation patterns are summarized. As ROS are important agents in antimicrobial process, the principles of piezocatalysis and ROS stimulation are also expounded Subsequently, the underlying mechanisms of bacterial death from piezoelectric materials are classified, including biofilm structure destruction, cell membrane permeate, energy metabolism interruption and immune regulation. This review also provides an in‐depth summary of the latest research progress of piezoelectric biomaterial, with an emphasis on their design strategies and working mechanisms for antibacterial purposes. Finally, the primary obstacles and future perspectives related to piezoelectric biomaterial are proposed and prospected.

## 2 Piezoelectricity and Piezocatalysis

The term “piezoelectricity” comes from the Greek words “piezein” (meaning “to press”) and “ēlektron” (meaning “amber”), indicating electricity generated by pressure.^[^ ^25^ ^]^ The piezoelectric property results from the offset of positive and negative charge centers (i.e., the formation of electric dipoles) of this non‐centrically symmetrical structure upon mechanical force. The electric dipoles then endow the piezoelectric materials with certain catalytic capacity. The wide application range of piezoelectric materials stems from their unique structure and adaptable catalytic performance.

**Figure 2:**
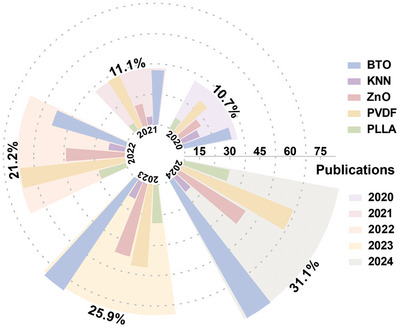
Statistical chart of the number of publications in the biomedical and antibacterial field in the last five years, represented by five piezoelectric materials.

To investigate the development potential of piezoelectric materials in biomedical applications, particularly in antibacterial treatment, we analyzed the publication data on piezoelectric materials applied for antibacterial or tissue regeneration (represented by barium titanate, potassium sodium niobate, zinc oxide, polyvinylidene fluoride, and poly‐l‐lactic acid) reported in the Web of Science over the past five years. The annual publication counts for each piezoelectric material, as well as the ratio of the total annual publications for these five materials to the overall publication total over the five‐year period, are presented in **Figure** **2**. The statistical findings indicate an upward trend in annual publications on piezoelectric materials, particularly highlighting a significant interest in those based on polyvinylidene fluoride and barium titanate.

### 2.1 Classification of Piezoelectric Materials

Generally, piezoelectric biomaterial consists of multiple components with meticulous design, among them the components that harness piezoelectric performance are mainly categorized into piezoceramic, 2D piezoelectric materials and piezopolymer. Their piezoelectricity is closely related to their lattice or molecular structures.

#### 2.1.1 Piezoelectric Ceramics

Piezoelectric ceramics are a series of inorganic polycrystalline materials and have high piezoelectric coefficients.^[^ ^26^, ^27^ ^]^ One of the most widely used piezoceramics was lead‐zirconium‐titanate (Pb(Zr, Ti)O_3_, PZT), featuring a typical perovskite crystal structure.^[^ ^28^ ^]^ Perovskite crystal structure is a famous type of crystal structure to possess piezoelectricity and ferroelectricity. The chemical formula denotes as ABO_3_, where A‐site ions are alkaline earth or rare earth elements with a 12 coordinated structure located and B‐site ions are usually transition metal elements in the 3d, 4d, and 5d groups, forming octahedral coordination with six oxygen ions.^[^ ^28^ ^]^ Non‐centrosymmetric changes to the ABO_3_ perovskite structure are essential for piezoelectric behavior, achieved through alterations in unit cell axes. Nevertheless, PZT is rarely applied in biological settings due to its cytotoxicity associated with the presence of toxic Pb^2+^ ions, thus lead‐free piezoceramics have become an area of active research interest.^[^ ^29^, ^30^ ^]^

**Figure 3:**
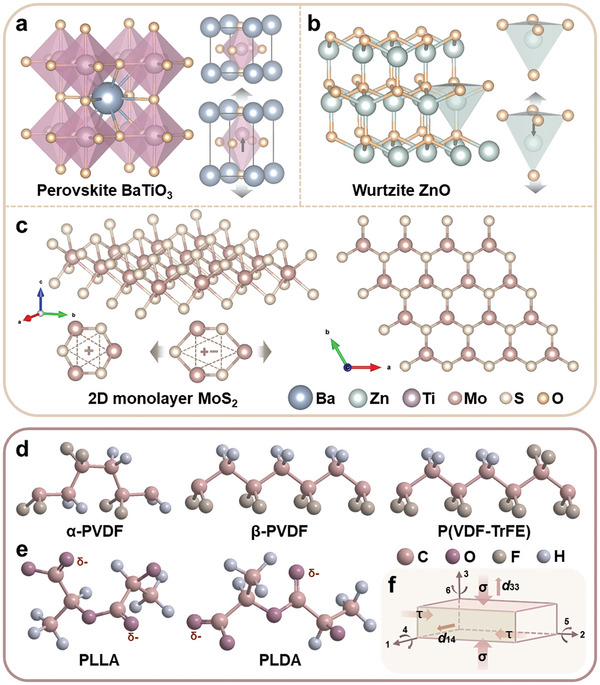
The atomic structures of piezoelectric materials: a) Perovskite BaTiO_3_, b) Wurtzite ZnO, c) 2D monolayer MoS_2_, d) PVDF, e) PLLA; f) Schematic of the relationship between force direction and electrical response.

Lead‐free piezoceramics are typically classified into three main categories: perovskite structure, bismuth layered structure, and tungsten bronze structure. Among these, perovskite‐based lead‐free ceramics, such as barium titanate, potassium sodium niobate, and sodium bismuth titanate along with their derivatives, are particularly noteworthy due to their superior piezoelectric performance and promising prospects for biological applications.^[^ ^31^ ^]^ Barium titanate (BaTiO_3_, BTO) was discovered in 1946 and was firstly used for bone repair in 1972. BTO has a high piezoelectric constant (*d*_33_ = 191 pC N^−1^) and low dielectric loss with outstanding biocompatibility, making it an early studying hotspot. The flexible movement of Ti^4+^ within the tetragonal unit cell enables the strong electrical polarity ability of BTO (**Figure** **3**a). Likewise, potassium sodium niobate ((K, Na) NbO_3_, KNN) is another lead‐free piezoceramic candidate with high piezoelectric constant (*d*_33_ = 80–120 pC N^−1^) but its biomedical research commenced relatively late. The modification of KNN through different methods and its phase boundary construction contributes largely to it piezoelectricity promotion.^[^ ^32^ ^]^ For example, KNN is compatible to dope many metal elements like Fe, Mn, and Co to introduce additional functionalities.^[^ ^33^, ^34^, ^35^, ^36^ ^]^ Lithium doped KNN (LKNN) is reported to perform better strength and piezoelectric properties (*d*_33_ = 235 pC N^−1^), which makes it preferable for electrophysiological osteogenesis and bone substitution.^[^ ^37^ ^]^

Bismuth layered materials are a type of lead‐free piezoelectric material with a complex layered structure, typically composed of alternating layers of alternating perovskite layers and (Bi_2_O_2_)^2+^ layers, stacked regularly along the *c*‐axis.^[^ ^38^ ^]^ Bismuth‐layered materials possess a unique crystal structure with rich atomic coordination and an attractive hybrid electronic band structure, which enables them to be highly adaptable in crystal structure design, with precise control over defect modifications and bandgap tuning.^[^ ^39^ ^]^ Bismuth‐layered materials show promise in various applications, including efficient catalysis, energy conversion, and are attracting growing attention in biomedical research.^[^ ^40^, ^41^, ^42^, ^43^ ^]^

Tungsten bronze structure, with the formula [(A1)_2_(A2)_4_C_4_][(B1)_2_(B2)_8_]O_30_, are widely studied for energy storage in dielectric capacitors.^[^ ^44^ ^]^ Their structure features a corner‐sharing network of B₁O₆ and B₂O₆ octahedra, forming tunnels of various shapes (pentagonal, quadrilateral, and triangular). Tungsten bronze structures are highly flexible, but this inherent structural complexity negatively impacts their energy storage performance and other properties, thus limiting their widespread application.^[^ ^45^ ^]^ Previous research about tungsten bronze mainly focuses on doping and coupled relaxor design to enhance their energy storage performance.^[^ ^46^, ^47^, ^48^ ^]^

#### 2.1.2 2D Piezoelectric Materials

Some binary piezoelectric materials with 2D structures exhibit advancement in tailoring flexibility and offer promises in many fields including sensing, catalysis, energy harvesting and so on.^[^ ^49^ ^]^ Zinc oxide (ZnO) is a binary piezoceramics with wurtzite structure, which belongs to a hexagonal crystal system in type of AB composition. As shown in Figure 3b, the structure of ZnO is alternating planes composed of tetrahedrally coordinated Zn^2+^ and O^2−^ ions packing along the *c*‐axis, in which dipole moments and polarization always occur.^[^ ^50^ ^]^ Its exceptional performances in electron mobility, photoelectric properties, and biocompatibility make ZnO highly appealing for biomedical applications, such as biological imaging probes and tissue engineering scaffolds.^[^ ^51^ ^]^ Due to their high specific surface area and tunable mechanical properties, 2D ZnO materials, particularly monolayer or few‐layer ZnO, show great promise for biomedical applications like biosensors, drug delivery systems, and tissue engineering, offering enhanced performance in biocompatible and bioactive environments.

Piezotronics in 2D materials was first confirmed experimentally in 2014 by the Hone and Wang groups, who detected piezo‐voltage and output current only from MoS₂ flakes with an odd number of atomic layers.^[^ ^52^ ^]^ Before this, 2D transition metal dichalcogenides (TMD) materials had already attracted significant attention due to their exceptional electrical and optical properties, with MoS₂ being one of the most extensively studied materials in the family. 2D piezoelectric MoS_2_ is also widely investigated in biomedical applications due to its modification flexibility and important roles of Mo and S element in enzymes and cell.^[^ ^53^ ^]^ The non‐centrosymmetric structure of top view is a convictive explanation for its piezoelectricity. MoS_2_ belongs to monolayer transition metal dichalcogenides due to the strong Mo−S covalent bonds within the monolayer, but weak van der Waals forces between layers (Figure 3c).^[^ ^54^ ^]^ This special structure enables MoS_2_ large relative surface area and high flexibility. Besides, being a polyvalent transition‐metal element, Mo enhances catalytic activities through electron transfer and valence changes, establishing MoS_2_ as a favored material in diverse fields like environmental science, catalysis, sensing, energy, and biomedicine.^[^ ^55^, ^56^ ^]^

Despite the excellent piezoelectric properties, piezoelectric ceramics and 2D piezoelectric materials suffer from poor toughness and distortion resistance, which severely hinders their biomedical applications, particularly as flexible implantable devices. To overcome this inherent shortcoming, they are always synthesized as functional nanoparticles and incorporated as dopants into the matrix or loaded as a coating on stiff scaffolds to endow whole materials with electroactivity.

#### 2.1.3 Piezoelectric Polymer

Many natural organisms are found to possess piezoelectricity, such as collagen, chitosan, keratin, and cellulose, and most of them have a highly aligned structure. Similarly, synthesized polymers like PVDF (polyvinylidene fluoride), PLLA (poly‐l‐lactic acid), PHB (polyhydroxy butyrate) possess piezoelectricity when molecular dipoles reorienting under external stimulation.^[^ ^57^ ^]^ The piezoelectricity of polymer is inferior to piezoceramics because of the high molecular weight and complex chain structure. The long‐range ordering of macromolecule structures makes the dipoles orientation less regular in comparison to lattice structure of piezoceramics.^[^ ^58^ ^]^ Nevertheless, piezoelectric polymer shows enhanced biocompatibility and degradation capacity. Additionally, piezoelectric polymers offer the advantage of versatility in processing methods under mild temperatures so that it can be processed into delicate structures and certain shapes though some elaborate processing technologies like 3D printing and electrospinning.

PVDF is one of the most widely explored piezoelectric polymers, which has five crystal phases due to the different alignments of H and F along the C–C backbone. Among these phases, α and β phases are the most common.^[^ ^57^ ^]^ The molecular chains are antiparallel and have no dipole moment in α phase resulting no piezoelectricity, while in β phase the dipole is parallel and have all‐trans conformation making it the main source of piezoelectricity (Figure 3d). The superior piezoelectric coefficient of all‐trans conformation PVDF is up to 20 pC N^−1^.^[^ ^59^ ^]^ Furthermore, it is available to alter the piezoelectricity through modulating β phase of PVDF by different parameters of process and introduction of nanoparticles.^[^ ^60^ ^]^ P(VDF‐TrFE) is a success case (30 pC N^−1^), where the copolymerization of tetrafluoroethylene (TeFE; –(CF_2_–CF_2_)–) with PVDF(–(CH_2_–CF_2_)–) introduce extra fluorine atoms leading to additional steric hindrance, thus restricting the formation of the α phase.^[^ ^61^ ^]^ With improved flexibility, residual polarization and electromechanical coupling factor, P(VDF‐TrFE) is highly competitive for application in tissue engineering scaffolds and flexible biosensors.

In the case of PLLA, whose monomer is a chiral molecule of lactic acid. Different arrangement of lactic acid and chiral isomers result in different configurations, but studies shows that only PLLA shows piezoelectricity with the conformation of levorotatory lactic acid monomers aligning in homogeneous way.^[^ ^62^ ^]^ The stable configuration of PLLA is described as left‐hand helix orientation, which is similar to many natural piezoelectric polymers like collagen.^[^ ^63^ ^]^ The helically orientation comes from the acyls (–CO–C–) alone the backbone. Under shearing in direction of parallel to polarization, the re‐orientation of acyls gives rise to the piezoelectric effect of PLLA (*d*_14_ = −9.82 pC N^−1^), which is different from other piezoelectric materials responsive to tension or pressure, as depicted in Figure 3e,f.^[^ ^64^, ^65^ ^]^ This unique shear piezoelectricity of PLLA together with its outstanding biocompatibility motivate its utilization in biomedical field. Take bone tissue engineering as an example, the shear piezoelectricity of PLLA is quite similar to the bioelectric environment created by the uniaxial alignment structure of collagen in bone.^[^ ^66^ ^]^

Comparing to piezoceramics, piezopolymers are advantageous in toughness, flexibility, and biocompatibility, but possess a relative weaker piezoelectric coefficient. These characteristics determine their secondary roles as functional components within piezoelectric biomaterial, while also leading to their frequent utilization in biosensing and detection devices.^[^ ^67^ ^]^ As for tissue engineering, the integration of piezopolymers and piezoceramics is preferable, with expanded potential to synergistically consolidate the piezoelectricity, biocompatibility and mechanical properties. In such piezoelectric composites, piezopolymers always pose as continuous phase while piezoceramics act as dispersed one.

Apart from conventional piezoelectric polymers, various piezoelectric artificial supramolecular have been investigated. Artificial supramolecular materials self‐assemble into non‐centrosymmetric multi‐level structures through the stacking of monomers or primary units, driven by non‐covalent interactions. These materials show potential for achieving piezoelectric effects comparable to those of conventional piezoelectric materials. For example, the artificial N‐terminally acetylated amino tryptophan polycrystalline film is reported to generate sufficient power to illuminate an LED.^[^ ^68^ ^]^ Cage‐like molecules can be constructed in a non‐centrosymmetric packing arrangement by utilizing crystal engineering to achieve hierarchical self‐assembly of asymmetrical cages.^[^ ^69^, ^70^ ^]^ With appropriate design and fabrication, they can be applied in energy‐harvesting systems. Metal–organic frameworks (MOFs) can also be designed to possess piezoelectric properties through non‐centrosymmetric structures. These materials are typically employed as functional components in piezoelectric biomaterial and have been shown to significantly enhance catalytic efficiency. UiO‐66 is one of the first MOF materials reported to demonstrate piezoelectricity in 2019.^[^ ^71^ ^]^ Subsequent research on piezoelectricity has extensively focused on UiO‐66, particularly the UiO‐66‐NH2 (Hf) variant, due to its enhanced piezoelectricity and catalytic activity.^[^ ^72^, ^73^ ^]^ Other piezoelectric MOF materials have been gradually reported and are being explored for biomedical engineering applications.^[^ ^74^, ^75^ ^]^

### 2.2 Activating Piezoelectric Response

The electromechanical coupling of piezoelectric implantation can be achieved through cellular traction and daily physical movements.^[^ ^19^ ^]^ A relatively stronger stimulation is required for exceptional functions of antimicrobial and antitumor processes in general. Thus, there is an urgent need for the prevalence of external stimulation, especially noninvasive methods. For bulk piezoelectric materials, the direct corona polarization treatment is a basic and necessary preliminary treatment for the dipole orientation within the dense and continuous structures. However, such corona treatment is avoidable in most piezoelectric biomaterial so far because piezoelectric components always exist as 0D particles or 1D fiber in isolated forms. In fact, in the design of piezoelectric biomaterial, noninvasive stimulations are preferable for their convenience and adjustability as well as a high upper limit of piezoelectric effect.

Infrared light has been recognized as an effective method for combined use with piezoelectric materials in infrared sensors and infrared imaging. In 2016, Wang et al. assembled TiO_2_ on ZnO to create a piezoelectric semiconductor‐based hybrid photocatalysts. In this heterojunction, the photochemical reactivity was enhanced due to the piezoelectric electric field generated in ZnO upon light absorption, which facilitated the separation of photogenerated electrons and holes.^[^ ^76^ ^]^ However, due to its poor penetration ability and unavoidable photo toxicity,^[^ ^77^ ^]^ this combination is mainly confined to skin regeneration and wound healing.^[^ ^19^ ^]^ By comparison, ultrasonic (US) shows high tissue penetrability and directed orientation ability, which are suitable for deep tissue therapy like bone and cartilage.^[^ ^78^ ^]^ There are two main physical effects occurring when US transfer: mechanical effects and thermal effects. The main mechanism for US mechanical effects lies in the bubble cavitation, which involves the pressure from collapse of tiny bubbles generated by US. The pressure is modified as Rashwan et al. pointed, that is up to a certain limit, the lower frequency of US is, the longer it will take for the periodic, thus more time for bubbles growing, which will enable bubbles to generate higher pressure when collapse.^[^ ^79^ ^]^ Wang et al. substantiated that US irradiation is a powerful and convenient approach for inducing polarization of inert PTFE into piezoelectric electrets, exhibiting much higher piezoelectricity than traditional piezoelectric materials.^[^ ^80^ ^]^ Thermal effects is unavoidable side effects of US, but several experiments have proved that it will not be harmful for therapeutic effect but US should be given to an appropriate extent.

Although piezoelectric materials do not inherently respond to magnetic fields, combining them with magneto strictive materials, such as CoFe₂O₄, provides an indirect yet feasible solution for magnetic responsiveness.^[^ ^81^, ^82^ ^]^ This combination retains advantages such as noninvasiveness, deep tissue penetration, and remote control, expanding the potential applications of these materials, particularly in medical devices and therapeutic materials.^[^ ^83^ ^]^ Such indirect stimulation can be achieved through other pathways, for example, microorganisms have been considered as a cutting‐edge external stimulation. In 2022, Tang et al. reported a material‐assistant micro‐organism strategy applied in piezoelectric materials study.^[^ ^84^ ^]^ They adopted *Bdellovibrio bacteriovorus*, which could prey on the Gram‐negative bacteria and exhibit rapid swimming and high‐speed rotation. The *Bdellovibrio* could generate mechanical stimulation through random collision for ZnO nanorods. This combination can sweep almost all the biofilms, much more effective than common antibiotics Metronidazole. Besides, the presence of fluid‐filled channels surrounding the ZnO nanorods provides ample oxygen for *Bdellovibrio*, forming positive feedback to some extent, which makes ZnO@Bdello more aggressive in biofilm removement. They also testified the blood biochemistry and serum routine tests of the animal after 24h implantation and validated its biocompatibility.

### 2.3 Piezoelectric‐Induced ROS

Reactive oxygen species (ROS) refer to a series of unstable, reactive, partially reduced oxygen derivatives, including hydrogen peroxide (H_2_O_2_), superoxide anion (·O_2_^−^), singlet oxygen (^1^O_2_) and hydroxyl radical (·OH).^[^ ^85^, ^86^ ^]^ They are highly reactive oxygen‐containing molecules. The strong reactivity derives from the existence of active unpaired electrons in their electronic configurations, according to molecular orbital theory.^[^ ^87^ ^]^ ROS generation can derive from normal metabolism such as aerobic respiration by‐product and also serve as self‐protection mechanisms to react abnormal status like bacterial invasion through oxidative stress.^[^ ^88^, ^89^ ^]^ However, over‐produced ROS may lead to inflammation and cellular oxidative damage. Thus, stimulating the production or consumption of ROS is crucial for maintaining a normal cellular microenvironment.

To date, a variety of ROS‐mediated therapeutic approaches have been extensively refined including photodynamic therapy (PDT), sonodynamic therapy (SDT), enzyme dynamic therapy, chemodynamic therapy (CDT), and more.^[^ ^90^ ^]^ Piezoelectric materials are well‐suited for these therapies because they are responsive to external stimulations like sound and light. According to the collected studies, piezoelectric materials can serve as a lever to balance the tissue microenvironment, producing ROS to defend against bacterial invasion or elimination ROS to thwart inflammation. Especially, ROS generation ability is a significant weapon for piezoelectric materials to sterilization. Here are possible pathways for ROS generation encompassing piezoelectric catalysis and nanozymes.

#### 2.3.1 Piezoelectric Catalysis

Piezocatalysis was first reported in 2010 that ZnO and BaTiO_3_ were able to catalyze water electrolysis under ultrasound exposure.^[^ ^91^ ^]^ The piezoelectric catalytic effect refers to the phenomenon where piezoelectric materials utilize their coupling of the piezoelectric effect and electrochemistry to facilitate redox reactions.^[^ ^92^ ^]^ It has been proved to serve as a powerful tool for utilizing the tunable electronic states to induce or accelerate chemical properties under stimulation.^[^ ^93^ ^]^ Piezoelectric catalysis is mainly manifested in piezoceramics, with limited occurrences reported in piezopolymers. These days, piezocatalytic effects have been rapidly employed in many medical purposes such as antimicrobial, antitumor, biomolecular detection, and so on.

As concluded by Liu et al. there are two popular theories for the explanation of piezocatalysis: energy band theory and screening charge effect.^[^ ^94^ ^]^ In energy band theory, piezoelectric effect generates a built‐in electric field that modulates the carrier density at interface, thereby altering the potential of valence band (VB) and conduction band (CB). This distortion of band structure is manifested as band tilting, which indicates a higher level of electron/hole separation and higher reactivity.^[^ ^95^ ^]^ There are two simplified models of band‐tilting due to the material intrinsic properties. For materials with narrow bandgap, carriers stay in a low energy level so that cannot afford to participate in redox reactions. The built‐in electric field promotes to positivizing the VB energy level and negativizing the CB, facilitating the involvement of electrons from CB in reduction reactions and the participation of holes from VB in oxidation reactions.^[^ ^15^ ^]^ The other situation concerning materials with wide bandgap, requires much energy for electron activation from VB to CB. In this scenario, the band bending leads to VB and CB getting closer, making electronic transitions easier to promote ROS production. The practical band tilting can be a more intricate process, primarily influenced by the intrinsic properties of the material.

**Figure 4:**
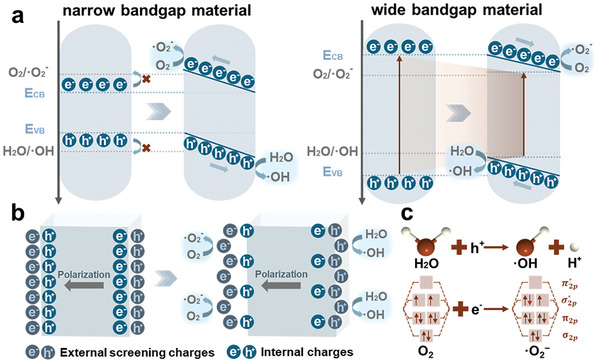
Schematic diagram of piezocatalysis: a) energy band theory; b) screening electron theory; c) molecular formula of O_2_/·O_2_^−^ and H_2_O/·OH redox reaction.

Different from that, the screening charge effect considers the piezoelectric potential to be the driving force of the reactions. It is the screening charges attracted on the polarized surface that involve the reaction. The greater the piezoelectric potential, the more screening charges are attracted, leading to more intense reactions.^[^ ^96^ ^]^ The redox reactions that occur through the piezoelectric catalysis mainly depend on the potential of conductive band (E_CB_) and valence band (E_VB_), which are material properties. Two redox pairs that are highly frequently discussed are O_2_/·O_2_^−^ and H_2_O(OH^−^)/·OH. Their voltages of the Half‐cell in an Electrochemical reaction (VHE) are close to the E_CB_ and E_VB_ of numerous piezoelectric materials separately. **Figure** **4** provides a schematic representation of these two reactions based on both theories.

These two mechanisms share a common feature of the tight relationship between piezoelectric potential and redox reaction but differ in the inherent action modes as well as the reactive charges carriers.^[^ ^94^ ^]^ The energy band theory conveys that the piezopotential adjusts the energy band level and helps with reaction occurrence. The internal charges of piezoelectric materials serve as the carriers. However, in the screening charge theory, piezopotential directly determines whether the redox reaction happens, and the charge carriers are external screening charges. The underlying mechanisms of piezocatalysis are still widely discussed but have not reached a clear conclusion (**Table** **1**). Both theories provide rational explanations drawing on their respective core concepts and experimental outcomes.^[^ ^97^ ^]^ Recently, Bößl et al. employed three piezoelectric catalysts with distinct energy band levels and piezoelectric properties to examine their capability to produce ROS and degrade Rhodamine B. The experimental results indicate that both mechanisms appear to be happening concurrently.^[^ ^98^ ^]^ It is noteworthy that due to the shape limitation of piezoelectric material, screening charges theory is not applicable to most piezoelectric biomaterial. Therefore, it seems that band theory is more popular and proficient in piezocatalytic studies with capable characterization methods, i.e., UV diffuse reflection to visualize the band tilting. Density function theory (DFT) calculation is also a powerful tool to mimic the electronic energy distribution and charge transfer pathways. Common calculation simulation includes projected density of states (PDOS), charge density difference plots and Bader charge estimates, etc. Nevertheless, some studies still employ the screening charge theory. In 2023, Shu explained the piezo‐photocatalytic mechanisms of Ag nanowires@BaTiO_3_ by integrating the energy band bending theory for photocatalysis with the screen charge effect for piezocatalysis.^[^ ^99^ ^]^ However, they merely proposed the existence of screen charges but did not explicitly validate their presence in the study.

**Table 1:** piezocatalytic effect explanations for piezoelectric materials and the relevant measurement and results.

Piezoelectric component	Stimulation form	theory	measurement	results	Ref.
MoS_2_/Cu_2_O	Ultrasonic	Energy band theory	UV–vis spectra	Energy gap decreased but did no specific numbers provided	[89]
Ag NWs@BTO	Ultrasonic and light	Energy band theory and Screen charge theory	Ultraviolet photoelectron spectroscopy (UPS)	Work Function: decrease from 4.88 to 4.84 eV	[99]
			UV–vis absorption spectra	Energy gap: decrease from 3.18 to 2.76 eV	
Al‐doped SrTiO_3_/TiO_2_	Ultrasonic	Energy band theory	UV–vis diffuse reflectance spectra	Energy gap: decrease from 3.14 to 3.07 eV	[100]
sulfur‐doped BTO	Ultrasonic	Energy band theory	UV–vis adsorption spectra Kubelka–Munk function	Energy gap: decrease from 3.02 to 2.48 eV	[101]
TiO_2_/BTO/Au	Light	Energy band theory	Not mentioned	Energy gap: decrease from 3.20 to 3.0 eV	[102]
KNN	Ultrasonic	Energy band theory	UV–vis diffuse reflectance spectra	Energy gap: fluctuate from 3.42 to 3.38 eV and then 3.61 eV	[103]
			XPS	2.17, 2.11, and 2.08 eV,	
HNTM/MoS_2_	Ultrasonic	Energy band theory	UV–vis diffuse reflectance spectra Kubelka−Munk function	Energy gap: decrease from 1.79 to 1.77 eV	[104]
PtRu/C_3_N_5_	Ultrasonic and light	Energy band theory	Tauc plots	Energy gap: decrease from 1.84 to 1.51 eV	[105]
black phosphorus/V_2_C MXene	Ultrasonic	Energy band theory	DFT calculations	Work Function: decrease from 4.559 to 3.839 eV	[106]

#### 2.3.2 Piezoelectric Nanozymes

Piezoelectric materials represent a promising new category of nanozymes, as their active ion components or vacancies can serve as reactive sites in catalytic reactions. Nanozymes are nanomaterials with biocatalytic capabilities, combining the high catalytic activity of natural enzymes with the stable physicochemical properties of nanomaterials.^[^ ^101^, ^102^, ^103^ ^]^

Recent related studies mainly focus on investigating intrinsic catalytic capabilities of nanomaterials as an attractive application field. To date, many nanomaterials have been recognized as remarkable enzyme‐like activities, such as Au nanoparticles, graphene oxide, CeO_2_, and MnO_2_ nanoparticles etc.

The discovery of POD‐like activity of Fe_3_O_4_ nanoparticles in 2007 open the era of research on natural enzymes alternative.^[^ ^107^ ^]^ Nanozymes circumvent the inherent drawbacks of natural enzymes for high expenses, limited stability, and challenges in recycling, while offering additional benefits like gentle reaction conditions, durability, and being well‐suited for mass production, which have been widely used in medical and biological fields.^[^ ^108^, ^109^ ^]^ The majority of nanozymes have the ability to mimic the functions of oxidoreductase enzymes like peroxidase (POD), catalase (CAT), oxidase (OXD), and superoxide dismutase (SOD). POD typically catalyzes substrate oxidation by decomposing H_2_O_2_ into H_2_O, which just suits the H_2_O_2_‐riched infection and tumor microenvironment. POD‐like activity is typically exhibited by variable valence metals and their oxides, which is responsible for Fenton‐like reaction.^[^ ^110^ ^]^

(1) $$H_{2}O_{2}+e^{-}\to OH^{-}+\mathrm{OH}$$

CAT nanozymes can catalyze the decomposition of H_2_O_2_ into O_2_ and H_2_O molecules, relieving the hypoxia of cells and protecting cells from oxidative stress.

(2) $$2H_{2}O_{2}\to 2H_{2}O+\cdot O_{2}$$

OXD reaction usually happens in the presence of oxygen, reducing it to H_2_O or H_2_O_2_. Generating an appropriate amount of H_2_O_2_ can help eliminate pathogens and exhibit antibacterial effects.

(3) $$O_{2}+e^{-}\to \cdot {O_{2}}^{-}$$

**Figure 5:**
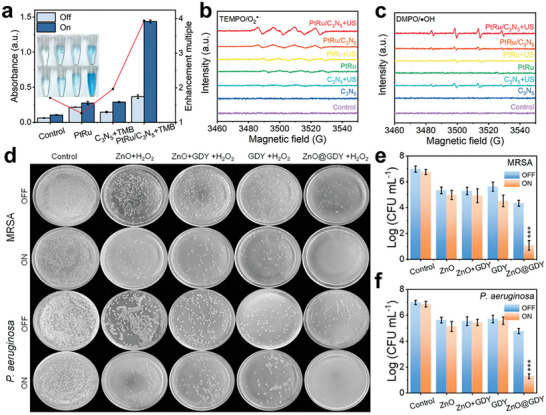
a) the OXD‐like activity of different piezoelectric groups; b) ·O_2_^−^ and c) ·OH trapped under different conditions. Reproduced with permission.^[^ ^105^ ^]^ Copyright 2022, John Wiley and Sons. d) digital photographs and counts of the number of colonies of e) MRSA and f) *P. aeruginosa* cultured on different materials. Reproduced with permission.^[^ ^114^ ^]^ Copyright 2022, American Chemical Society.

Recently, nanozyme antibacterial therapy (NABT) has emerged recently, offering the benefits of highly effective broad‐spectrum antimicrobial activity against MDR strains, minimal bacterial resistance, and low cytotoxicity.^[^ ^111^, ^112^ ^]^ Yu et al. elucidates the mechanisms of piezoelectric effect to permit nanozymes to exhibit catalytic activity.^[^ ^113^ ^]^ Piezoelectric materials are rarely employed as nanozymes alone due to the relatively low enzymatic performance unless they are assisted by external stimulation or undergo modification. When exposed to stimulation such as US or NIR, a built‐in piezoelectric field is established, modulating carrier migration. This efficiently separates and transfers electron–hole pairs, leading to charge accumulation or a potential gradient at the material surface or interface. Since enzyme catalytic processes inherently rely on electron transport, this modulation of electron dynamics may influence electron transfer at the nanozyme's active site, thereby enhancing its catalytic activity. In addition, other nanozyme materials can be loaded onto piezoelectric surfaces to form heterostructures, where the piezoelectricity contributes to an interfacial electric field that promotes the directional flow of carriers between materials, transferring e−/h+ to the nanozyme materials and enhancing related enzyme‐catalytic activities. Shi et al. (2022) introduced a heterostructure of platinum–ruthenium nanoalloys decorated multivacant graphitic carbon nitride C_3_N_5_ nanosheets (PtRu/C_3_N_5_).^[^ ^105^ ^]^ The Schottky junction facilitates charge separation, enhancing the electron transfer process of OXD‐like activity, piezoelectric catalysis and H_2_ generation from photocatalyst. They tested the ·O_2_^−^ and ·OH production activity and found out that the OXD‐like activity of PtRu/C_3_N_5_ was a 3.9‐fold boost than solely C_3_N_5_ (**Figure** **5**a). The electron paramagnetic resonance (EPR) results indicate that PtRu/C_3_N_5_ exhibits superior activity compared to either single PtRu or C_3_N_5_. Moreover, exposure to ultrasonic (US) radiation further enhances the generation of ·O^2−^ and ·OH species, thereby augmenting the catalytic performance of the composite material (Figure 5b,c). In another study conducted by Bai et al., reported a heterostructure of ZnO nanorod and graphdiyne nanosheets, possessing POD‐like activity to decomposite H_2_O_2_ and generate ROS.^[^ ^114^ ^]^ This heterostructure nanozyme exhibits almost 100% antibacterial efficacy against methicillin‐resistant *Staphylococcus aureus* (MRSA) and *P. aeruginosa*, functioning as a potential nanozyme‐based skin patch for rapid wound disinfection (Figure 5d–f).

## 3 Configurations of Piezoelectric Biomaterial

For piezoelectric implants, the balance among piezoelectricity, biocompatibility and mechanical performances is a crucial consideration. The establishment of implants with piezoelectric functions is a critical aspect in the configuration of piezoelectric biomaterial. It is also a compelling research area that demands careful exploration and intelligent design.

### 3.1 Piezocatalysis Enhancement through Electronic Modulation

Piezoelectricity and ROS have made significant contributions to the enhancement of antibacterial effect. However, the concentration of free charges of perfect piezoelectric materials cannot meet the requirement of enough ROS generation for antibacterial purpose even with the external stimulations activated. Further modifications on microstructure to enhance the separation of charge carriers and appeal reactants adsorption are heated research interests in piezoelectric materials structure design. To date, many strategies have been developed to modify the microstructure of piezoelectric materials and further boost the antimicrobial effectiveness, including defect engineering and piezoelectric heterojunction foundation.

#### 3.1.1 Defect Engineering on Lattice Structure

The defects not only function as locations for molecular chemisorption, but also act as pathways for energy and electron transfer in a spatial sense (the reactive sites of redox reaction), which serves as a capable tool for increasing the piezoelectricity and piezocatalysis.^[^ ^115^ ^]^ In terms of piezocatalysis, the generation of ROS derives from the separation of electron–hole pairs. However, due to the facile recombination of electrons and holes, common piezoelectric materials exhibit relatively low quantum yields of ROS.^[^ ^116^ ^]^ The solutions enhancing charges separation and inhibiting rapid charges recombination are emphasized, which include defect engineering.^[^ ^22^ ^]^ Atomic vacancies induced by defects can facilitate the separation of e⁻/h⁺ pairs and promote the adsorption of O₂ and H₂O, thereby lowering their redox energy barrier.^[^ ^117^ ^]^ Oxygen vacancies are prevalent in metal oxides and semiconductors, probably because of the reactivity of the oxygen atom and the high content in the crystal lattice. As an important type of atomic vacancy in defect engineering, oxygen vacancies can be induced through various methods. For instance, enhancements in preparation techniques, such as sand‐grinding, can significantly increase the concentration of oxygen vacancies.^[^ ^118^ ^]^ Moreover, element doping shows connected relationships with modulating the formation of oxygen vacancies. Many studies have figured out that doping elements into piezoelectric materials could increase the local crystal asymmetry and adjust the charge density, which directly improved the piezoelectric properties.^[^ ^119^ ^]^

**Figure 6:**
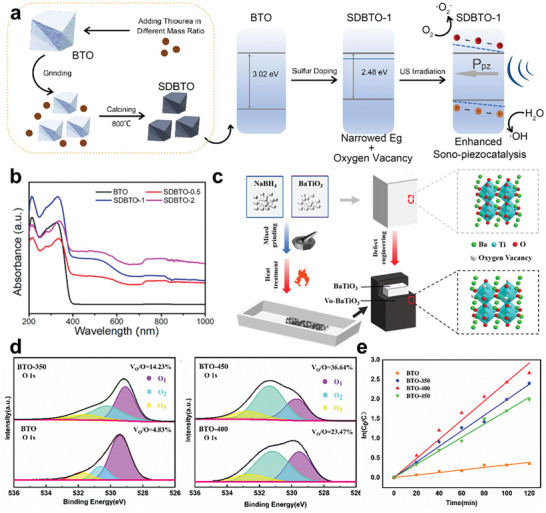
a) Scheme of sulfur doped BTO preparation and piezocatalytic mechanism; b) UV–vis adsorption spectrum of BTO with different sulfur doping degrees. Reproduced with permission.^[^ ^101^ ^]^ Copyright 2022, Elsevier. c) Synthesis process of Oxygen Vacancy‐Engineered BTO; d) O1s high‐resolution XPS spectra of different BTO; e) The first level kinetic reaction rate constant of Rh B degradation with modified BTO. Reproduced with permission.^[^ ^121^ ^]^ Copyright 2023, American Chemical Society.

While oxygen vacancies may have a detrimental effect on the overall macroscopic piezoelectric properties of the material, a mild presence of oxygen vacancies can significantly enhance the piezocatalytic activity. This is because the presence of electropositive oxygen vacancies restricted electrons and further impeded electron–hole recombination. As reported by Wang et al. (2022), they constructed a sulfur‐doped barium titanate piezocatalysis through ball mill grinding and annealing, transforming BTO into its tetragonal phase and introducing oxygen vacancies to enhance piezoelectric properties (**Figure** **6**a).^[^ ^101^ ^]^ They conducted the photo luminescence (PL) spectra of BTO doped with varying degrees of sulfur (SDBTO) to test the electron–hole combination efficiency (Figure 6b). It turned out to be in the sequence of SDBTO‐1 < SDBTO‐0.5 < SDBTO‐2 ≈ BTO (the numbers refer to the mass ratios of sulfur dopants: BTO), indicating both an excess and a scarcity of oxygen vacancies went against the reduction of electron–hole pair recombination. They summarized the mechanisms of SDBTO into three points: sulfur doping played crucial role in (1) serving to the augment the crystal asymmetry; (2) narrowing bandgap; and (3) inducing rational quantities of oxygen vacancies, collectively bolstering piezoelectric catalytic efficiency. Similar mechanisms of element doping regulate oxygen vacancy for piezocatalytic antibacterial treatment was also reported by Wei et al.in 2023.^[^ ^120^ ^]^ They found out that doping effectively enhanced polarization with an increased piezoelectric coefficient by nearly 2.6 times. In addition to piezoelectric responsiveness, Ce‐doping also gave rise to atomic vacancies, providing electron trapping sites and promoting the separation of e/h+ pairs from the band structure.

He et al. synthesized self‐doped barium titanate with controlled oxygen vacancy concentrations through NaBH4 thermal reduction method as shown in Figure 6c.^[^ ^121^ ^]^ NaBH4 is a strong reductant that undergoes decomposition under certain thermal conditions. The reduction products included active hydrogen that could capture the lattice oxygen to create the oxygen vacancy. This vacancy engineering regulated the oxygen vacancy concentrations by employing varying heating temperatures. The XRD, Raman spectroscopy, and XPS verified the existence of oxygen vacancy and lattice distortion (Figure 6d,e). This detect‐modified BaTiO3 exhibited significant ROS triggering ability for swift and effective sterilization under ultrasound stimulation.

#### 3.1.2 Heterojunction Construction

Piezoelectric materials, as members of the semiconductor family, are suitable candidates for heterojunction composition due to their outstanding electronic structure regulation and mechanical stability.^[^ ^122^, ^123^ ^]^ Heterojunctions refer to the junction of two dissimilar semiconductors with unequal energy band structures.^[^ ^124^ ^]^ The differences of two components provide a built‐in electric field at the interface, where carriers tend to shuttle between two semiconductors. This process tilts the energy band tilting and establishes new band alignment strengthening the feasibility and effectiveness in achieving the spatial separation of electron–hole pairs. Heterojunction construction with piezoelectric materials participation has been proved to be an effective approach to improve the charge separation efficiency.

**Figure 7:**
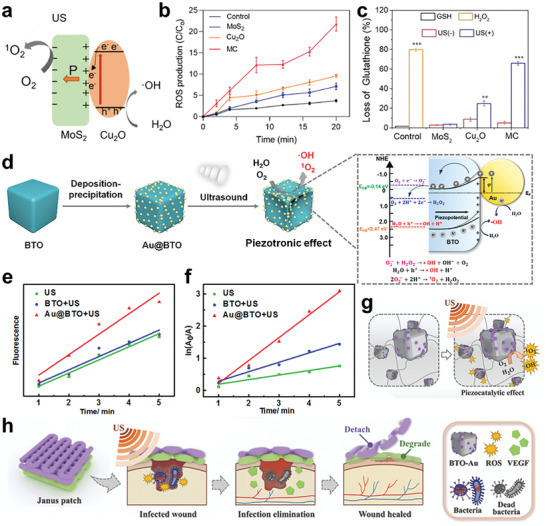
a) Scheme of MoS_2_/Cu_2_O heterojunction piezocatalytic mechanism; b) ROS production under US irradiation through DCFH fluorescence probe; c) Percent of Glutathione (GSH) loss under different conditions. Reproduced with permission.^[^ ^89^ ^]^ Copyright 2023, John Wiley and Son. d) Schematic illustration of Au@BTO nanocomposites preparation and sono‐sensitivity mechanism to generate ROS; e) Fluorescent intensity of ·OH from different samples under US stimulation; f) The rate constants for DPBF decomposition under different conditions. Reproduced with permission.^[^ ^127^ ^]^ Copyright 2021, Elsevier. g) Schematic illustration showcasing the piezocatalytic effect of BTO‐Au; h) Schematic illustration of the fabrication and antibacterial process of piezoelectric janus patch. Reproduced with permission. Reproduced with permission.^[^ ^126^ ^]^ Copyright 2023, American Association for the Advancement of Science.

In piezoelectric material and sonosensitizer heterojunction, piezoelectric materials serve as charges transfer station under polarization, which improve the charge transfer and promote the production of ROS. In 2023, Wang et al. developed piezoelectric MoS_2_ and Cu_2_O heterojunctions synthesized by hydrothermal method to treat infection through efficient SDT (**Figure** **7**a).^[^ ^89^ ^]^ The electron/hole separation enhanced upon US irradiation with MoS_2_. The electron enrichment give rise to the transition from O_2_ to ROS, while the hole enrichment acts as the receptor of electron from Cu_2_O, facilitating the oxide of ·OH. The reaction rate of ROS output from MoS_2_/Cu_2_O exceeded that of any single material group by at least 100% (Figure 7b). In this heterojunction, the coupling of MoS_2_ and Cu_2_O facilitates efficient charge transfer and promotes free electrons generation. Besides, Cu enables valence conversion from Cu (I) to Cu (II), thereby enhancing the oxidation of glutathione and simultaneously disrupting the bacterial antioxidant defense system (Figure 7c). Interestingly, it is always 2D piezoelectric materials such as MoS_2_, black phosphorus, C_3_N_5_ that take part in the heterojunctions, probably limited by the preparation methods and the requirement to maximize the size of the phase interface.^[^ ^104^, ^105^, ^106^ ^]^

Schottky junction is categorized as a heterojunction, built by connecting metal and semiconductors rather than two semiconductors. Schottky junctions are typically crafted using composite piezoelectric cubes, with piezoceramic materials featuring cell unit structures being favored for this purpose. BTO with Schottky junction modified by Au nanoparticles (Au@BTO) is a widely reported type.^[^ ^7^, ^125^, ^126^, ^127^ ^]^ It was firstly applied in biomedical field by Wu et al. in 2021. They studied the feasibility of Au@BTO used for piezoelectric catalysis and bacterial elimination.^[^ ^127^ ^]^ They explained that electrons flow towards BTO while holes move to Au at the interface. This exchange of carriers adjusts the energy levels of the valence and conduction bands, bringing them closer to the redox reaction site and thereby reducing the barrier for electronic transitions (Figure 7d). The ROS detection showed that the Au@BTO generate 1.63 times of ·OH under US and possess more of 1.42 times production rate in comparison to BTO (Figure 7e,f). Subsequently in 2023 Huang et al. fabricated this Au@BTO nanoparticle and co‐printing it with poly(ethylene glycol) diacrylate hydrogel forming 3D patches for wound healing (Figure 7g,h).^[^ ^126^ ^]^ The application of Au@BTO patches demonstrated a remarkable therapeutic effect on the dorsal full‐thickness skin wounds of *S. aureus* infected models within a 10‐day period.

### 3.2 Incorporation of Antibacterial Metal Ion

Certain essential trace elements are of great importance in physiological processes and some metal elements are proved to equip with antimicrobial ability, such as Ag, Cu, Zn, and Co, etc. The addition of metals can function as a conductive phase amplifying the intensity of the polarized electric field and simultaneously releasing metal ions with antibacterial properties.^[^ ^128^ ^]^ The intrinsic electric field between piezoelectric materials and bacteria could exert a discernible influence on the liberation of metal ions. The integration of metal components into piezoelectric materials is an advantageous method.

**Figure 8:**
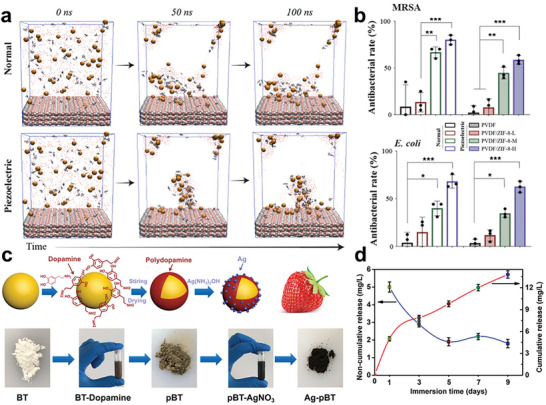
a) Molecular dynamic simulation comparation of Zn^2+^ dispersion in normal and piezoelectric conditions; b) The antibacterial rate after 24 h cultivation of MRSA and *E. coli*. Reproduced with permission.^[^ ^130^ ^]^ Copyright 2023, Elsevier. c) fabrication diagrams of Ag‐decorated barium titanate; d) The Ag ion concentration released and the cumulative value during 9 d soaking. Reproduced with permission.^[^ ^138^ ^]^ Copyright 2020, Elsevier.

Zinc (Zn) is a popular candidate for piezoelectric materials doping for its comprehensive effects of angiogenic, osteogenic, and antibacterial properties. Chen et al. have reported a PVDF/ZIF‐8 piezoelectric foam nanogenerator for bone regeneration.^[^ ^129^ ^]^ Zeolitic imidazolate framework‐8 (ZIF‐8), one of the metal‐organic frameworks (MOFs), is found to enhance the proton exchange and conductivity of PVDF membranes. Some studies have established that ZIF‐8 is a Zinc ionophore and a popular antibacterial agent, showing superiority over traditional ZnO.^[^ ^130^ ^]^ ZIF‐8 demonstrates gradual disintegration and sustained release of Zn^2+^ ions in body fluid environment, leading to better biological effect. Besides, PVDF tend to enrich Zn^2+^ gathering around and increasing local concentration through piezoelectricity (**Figure** **8**a). The MRSA and *E. coli* cultured experiments revealed the dependence on the antibacterial properties of ZIF‐8 concentration (Figure 8b). Similar effect has also been observed by Zhai et al., their Cu doped KNN was able to target and release Cu^2+^ acting on bacteria.^[^ ^131^ ^]^ They also confirmed that surface potential was the decisive factor of Cu^2+^ reaching bacteria. The KNNCu discs with 5kV external electric field polarization exhibited heightened antimicrobial efficacy in comparison to those under 3kV polarization and untreated samples.

Silver (Ag) has been considered as an effective antibacterial agent owing to its strong bactericidal activity and broad spectrum without inducing bacterial resistance. Ag in shapes of nanoparticles and nanofiber have been utilized in composite piezoelectric material designing.^[^ ^132^, ^133^ ^]^ Shuai et al. incorporated silver into the BTO surface by polydopamine coating modification. The in‐situ growth of Ag involved the integration of decorated Ag onto polydopamine, facilitated by the redox of silver ammonia ions and catechol or amino groups (Figure 8c). The silver ions (Ag^+^) can participate in superoxide anion generation in the presence of dissolved oxygen and protons (2H^+^ + O_2_ + 2e^−^ → H_2_O_2_; H_2_O_2_ + 2H^+^ + 2Ag→2Ag^+^ + 2H_2_O).^[^ ^134^ ^]^ This process can be further enhanced by the piezoelectric effect generated by the scaffold. The Ag^+^ release experiment showed a 13.8 mg L^−1^ cumulative Ag^+^ concentration in 9 d, falling within the inhibitory range of 10–100 mg L^−1^ against bacterial growth while preserving biocompatibility (Figure 8d).^[^ ^135^ ^]^

### 3.3 Piezoelectric Composite Materials

The previously approaches mentioned are chiefly conducted within the nanoscale domain for the purpose of enhancing piezoelectric and antibacterial characteristics. From a micro perspective of material design, these piezo nanomaterials can also be combined with other components, considering the requirements of practical tissue repair. Selection of matrices and scaffold construction techniques to incorporate piezoelectric material are of great importance (**Table** **2**).

Many of them mainly involved physical blending piezo nanomaterials with biocompatible materials. With respect to matrices or scaffolds, both synthetic polymers (such as PCL,^[^ ^106^, ^136^ ^]^ PLLA,^[^ ^137^ ^]^ PVDF,^[^ ^129^, ^132^, ^138^ ^]^) and hydrogels (including chitosan, gelatin, hyaluronic acid,^[^ ^105^, ^139^ ^]^ poly(ethylene glycol) diacrylate,^[^ ^126^ ^]^) are choices available. At the same time, tissue‐inducible ceramics of hydroxyapatite (HA), biphasic calcium phosphate (BCP), and bioactive glass are also appropriate options to be sintering together with piezoelectric materials.^[^ ^140^, ^141^, ^142^, ^143^ ^]^ These combinations are suitable for both hard and soft tissue infection. Besides, coating is another effective method which might be feasible for those high‐milting point materials like Ti alloy, targeting bone repair materials mostly.^[^ ^125^, ^144^, ^145^ ^]^

Meanwhile, for the nanocomposite, one of the most important difficulties is realizing highly dispersed piezoelectric nanoparticles. Prokhorov et al. prepared a hydroxylated BTO (OH‐BTO) which demonstrated good dispersion in chitosan matrix.^[^ ^146^ ^]^ According to FTIR result, they hypothesized that the side groups of chitosan and OH groups of BTO NPs collaboratively contribute to hydrogel bands and the homogeneous network for cell adhesion. The synergistic effect of piezoelectric NPs and CS contribute to the good biocompatibility, non‐toxicity, as well as high piezoelectric coefficient (*d*33 of 35 wt% OH‐BTO NPs in CS measured at 11.29 pC N^−1^), which is compatible with human bone (≈8 pC N^−1^). Piezopolymer fiber is another addictive to endow certain tissues requiring a little piezoelectricity, such as cartilage. Vinikoor et al. introduced an injectable piezoelectric hydrogel combining short nanofibers of PLLA and collagen matrix.^[^ ^147^ ^]^ The rhodamine B dyeing experiment and SEM verified the uniformly distribution of NF‐sPLLA in hydrogel. This piezoelectric hydrogel can be noninvasively inoculated into joint cartilage defects with irregular shapes to avoid invasive implantation surgery and severe cartilage defects for the treatment of Osteoarthritis.

**Table 2:** Design and fabrication of piezoelectric scaffold and the target tissue therapies.

Piezoelectric component	Materials design and technique		Therapy	Ref.
	Nanoparticle	Scaffold		
BTO	Au Schottky junction: redox reactions	1) Evaporate BTO solution on glass dish 2) Pour PCL solution into dish and evaporate solvent	Root canal infection	[7]
	Core–shell composites: BaTiO_3_ synthesize based on Ag Nano Wires	–	–	[99]
	Defect engineering: sulfur‐doped	–	Orthopedics	[101]
	Schottky junction: Au deposition	TiO_2_/BTO core/shell nanorod array	Wound healing	[102]
	–	Blend with OHA/THM‐APMH hydrogels	Wound healing	[139]
	Schottky junction: Au deposition	Coat on Ti	Orthopedics	[125]
	Au Schottky junction: redox reactions	Blend with PEGDA hydrogels	Would healing	[126]
	Schottky junction: Ag deposition	Blend with PEGDA/SF‐MA hydrogels	Would healing	[148]
	1) dopamine modification 2) Ag in situ growth: redox reactions	1) Mix with PVDF and fabricating 2) Selective laser sintering (SLS) technique	Orthopedics	[132]
	–	Mix with 45S5 bioactive glass	Orthopedics	[143]
	Berberine chloride encapsulation	–	Osteomyelitis	[149]
	–	Mix with clear resin	Oral anti‐adhesion	[150]
	1) Defect engineering: cerium doped 2) Core–shell composites: SiO_2_ nanoparticles and BTO coating	–	–	[120]
	–	Mix with gelatin methacryloyl	Periodontal disease	[151]
Barium calcium zirconate titanate (BCZT)	–	Mix with PCL	–	[136]
	Add cupric sulfate solution into BCZT and stir	1) 3D printed PEKK scaffold 2) Immerse and load PDA, HA, and BCZT particle on PEEK	Orthopedics	[152]
SrTiO3	Al^3+^ doped SrTiO_3_ Heterojunction: SrTiO_3_/TiO_2_	Modified on Ti	Dental implants	[100]
KNN	Defect engineering: copper doped	–	–	[131]
	–	1) Ball mill NKN and 1393BG 2) Prepare sample use cold isostatic press (CIP)	–	[142]
	–	Embed HA and KNN power into chitosan	Orthopedics	[153]
MoS_2_	Heterojunctions: MoS_2_ and CuO_2_	–	Wound healing	[89]
	Heterojunctions: MoS_2_ and porphyrin‐based hollow metal−organic framework and red blood cell membrane	–	Osteomyelitis	[104]
Bi_2_WO_6_	Heterojunctions: Bi_2_WO_6_ and TiO_2_	Modified on Ti	Orthopedics	[154]
ZnO	Heterojunctions: ZnO and graphdiyne nanosheets	–	Wound healing	[114]
ZnO/PVDF	–	1) Mix with PVDF/sodium alginate piezoelectric hydrogel 2) 3D printing technology	Wound healing	[155]
PVDF	–	Immers PVDF foam‐based sheet into ZIF‐8 suspensions	Orthopedics	[129]
	–	Electrospinning with Ag nanoparticle doped	Wound healing	[133]
PPy	–	Multilayers assemble with carbon nanotubes (CNTs) and carbon black‐doped PVDF‐HFP film	Wound healing	[12]
PLLA	–	Mix with collagen I rat tail (A1048301, Gibco)	Osteoarthritis (anti‐inflammation purpose)	[147]
black phosphorus (BP)	Heterojunctions: BP and V_2_C MXene	1) Blend with PCL 2) Electrospinning	Wound healing	[106]
C_3_N_5_	Heterojunctions: PtRu and C_3_N_5_	Embed heterojunction into hyaluronic acid	Wound healing	[105]

## 4 Antibacterial Theories of Piezoelectric Materials

The pathogen introduced during implantation along with the surrounding inflammation may lead to an infection or disrupt the host environment, making it more susceptible to infections.^[^ ^156^ ^]^ Infection microenvironment refers to both pathogens themselves and the immediate environment in which they grow and persist in infected sites of the hosts. Once the interaction between the host and pathogen progresses, both entities continually reshape the environment. The infectious microenvironment poses a significant challenge to traditional antibiotics. The swift colonization of bacteria leads to alterations in the pH of infected areas through hypoxic fermentation and enzyme secretion. The resulting acidic conditions and multiple secreted enzymes might deactivate the antibiotics.^[^ ^157^, ^158^ ^]^ Besides, the bacterial communities and their secretion extracellular polymeric substances (EPS) participate in the formation of biofilm, which is primarily composed of polysaccharides, proteins, and nucleic acids. EPS is the major source of antibiotic resistance and the development of chronic bacterial infections,^[^ ^159^ ^]^ because it acts as a physical barrier limiting the penetration and effectiveness of antimicrobial agents, while also promoting genetic exchange and adaptive responses within the biofilm. In addition, the immune response is also impacted by the surrounding microenvironment. For instance, in oxygen‐deprived environments, the bactericidal function of phagocytes may be compromised because of the lack of ROS.^[^ ^160^ ^]^ The polarization phenotype of macrophages is a significant weight for regulating ROS balances. M1 phenotype is crucial in engulfing pathogens, whereas the M2 phenotype is in charge of eliminating excess ROS from the environment and supporting tissue regeneration.

**Figure 9:**
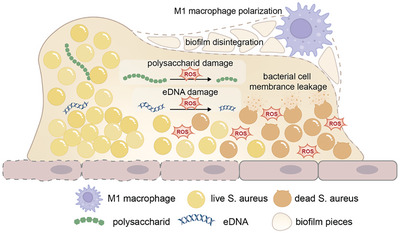
Schematic diagram of the destruction process of the infection microenvironment.

Piezoelectric materials strongly affect pathogens and their residential environment. The piezoelectricity and peizocatalysis donate activated electron to disturb the infectious environment and threaten the stable survival of bacteria. The reactive electron would participate in the process of interrupting stable electron transformation inside and outside of the bacteria. To achieve the goals of destroying the infection microenvironment and then deactivating bacteria, possible approaches aim at the structural stability of biofilms, bacterial membranes, catalytic activity, transporter activity, metabolic processes, and immune microenvironment, presenting in **Figure** **9**.

### 4.1 Interaction with Biofilm

**Figure 10:**
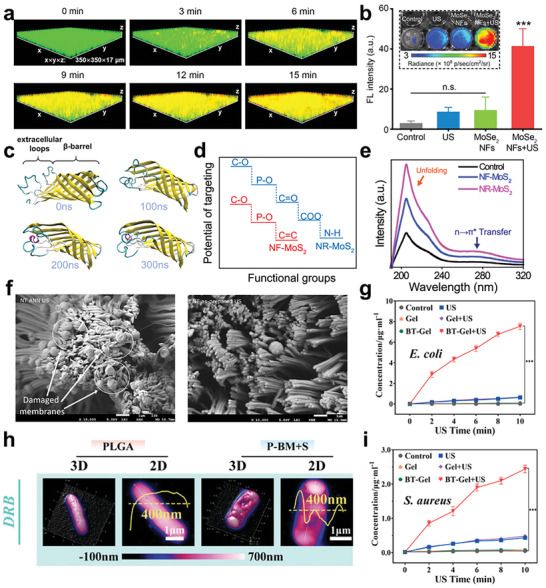
a) Penetration process of RhB‐labeled MoSe2 NFs in biofilms; b) Fluorescence intensity under different treatments. Reproduced with permission.^[^ ^164^ ^]^ Copyright 2023, American Chemical Society. c) The variation of OmpA protein 3D structure during molecular dynamic simulation under nanosecond‐scale pulsed electric fields. Reproduced with permission.^[^ ^166^ ^]^ Copyright 2023, Elsevier. d) Main result of 2D‐FTIR‐COS that reveal the biofilms functional groups and e) UV–vis absorption of biofilms under different MoS2 nanosheets treatment. Reproduced with permission.^[^ ^167^ ^]^ Copyright 2021, Springer Nature. f) SEM images of *S. epidermidis* bacteria on PLLA nanotexture before (right) and after (left) US simulation. Reproduced with permission.^[^ ^172^ ^]^ Copyright 2022, Royal Society of Chemistry. Concentration of g) *E. coli* protein and i) *S. aureus* leakage under different conditions. Reproduced with permission.^[^ ^139^ ^]^ Copyright 2023, Elsevier. h) 2D and 3D AFM morphologies and overall height of DRB contact with PLGA and P‐BM+US. Reproduced with permission.^[^ ^106^ ^]^ Copyright 2024, John Wiley and Sons.

Biofilms act as a physical barrier preventing antibiotics from penetration and taking effect by enzymatic decomposition or adsorption. For instance, anionic components of biofilm matrix such as polysaccharides and extracellular DNA can react with cationic antibiotics and deactivate them.^[^ ^161^ ^]^ Such mechanisms allow the antibiotic resistance of bacteria attaching to biofilm 1000 folds higher than that of planktonic bacteria.^[^ ^162^, ^163^ ^]^ Thus, the ability to swiftly penetrate biofilm is a crucial indicator of piezoelectric antibacterial materials. Gao et al. fabricated a nanoflower MoSe_2_ which possessed enzyme‐mimic activities.^[^ ^164^ ^]^ Study had shown that the MoSe_2_ NFs were able to penetrate the whole biofilm of MRSA within 15 min (**Figure** **10**a). The crystal violet staining and bacterial viability verified the anti‐biofilm and antibacterial effect (Figure 10b).

According to Xiao et al., EPS allows a high concentration of electron to transport in the space between cells and surrounding microenvironments.^[^ ^165^ ^]^ Therefore, it seems available to kill bacteria through disturbing the structure of biofilms and further interrupting the electron transportation. Zhao et al. found that *E. coli* EPSs were likely to be reduce serving as electron acceptors when they contact piezoelectric materials.^[^ ^166^ ^]^ After attaching to the wound, the PVDF‐TrFE films released the screening charges which destroy the molecular configuration. The molecular dynamics (MD) simulation revealed a notable conformational change in the extracellular loop region of outer membrane protein A (OmpA) in Figure 10c. Analysis of the root mean square deviation (RMSD) for OmpA residues 62–70 further elucidated a significant alteration in the secondary structure within this specific region. This was probably attributed to the disruption of hydrogen bonding, as indicated by the marked improvement in the RMSD of residues 63–67, suggesting a change in hydrogen bond length. Shi et al. examined the variations in biofilm components and demonstrated that polysaccharide intercellular adhesin and extracellular DNA are attacked in the electron transport of their piezoelectric nanosheets.^[^ ^167^ ^]^ Analysis using 2D‐Fourier transform infrared‐correlation spectra maps (2D‐FTIR‐COS) revealed significant changes in functional groups following interactions with nanohole‐MoS2, while UV–vis absorption spectroscopy indicated orientation transition and unfolding of protein structure (Figure 10d,e). In addition, Xu et al. pointed out that oxidatively deprive polysaccharides/NADH in biofilm was available to disturb electron transport chain and depressed biofilm energy metabolism.^[^ ^168^ ^]^ They loaded piezoelectric C_3_N_4_ nanosheets on hydrogel for diabetic foot wound healing. In their conception, US radiation induces electronic transitions that generate highly oxidative holes, serving as predators for polysaccharides/NADH, while also permeating H_2_ from the reduction side, thereby contributing to biofilm eradication. The respective roles played in the anti‐biofilm process have been independently validated through hole‐ and electron sacrificial agents.

### 4.2 Increased Bacterial Membrane Permeability

The bacterial surface is negatively charged mainly because of the phosphoric acid and carboxyl functional groups on the membrane.^[^ ^13^ ^]^ Therefore, bacteria tend to be attracted to the “pole+” surface of piezoelectric materials, and then the neutralization of negative charges and positive charges would alter the structure of lipid bilayer, leading to the bacterial cell membrane penetration.^[^ ^169^, ^170^ ^]^ The mechanism probably lies in the coulombic interaction of positive charged surfaces and negative charged cell membrane, resulting in the increased permeability of bacterial membrane and eventually the lysis of bacteria.^[^ ^137^ ^]^ This mechanism is frequently reported in piezoelectric polymers such as PLLA or PVDF, because unlike piezoceramics, the piezoelectricity of piezopolymer is too weak to hydrolyze water to produce ROS. As reported by Ando et al. the possible reasons for PLLA antibacterial effects were electroporation owing to high‐voltage output, electric current, rather than ROS.^[^ ^171^ ^]^ When an electric field with a potential difference of more than 1V applies to the bacteria, the micro‐electric environment instability of membrane may form pores, causing bacterial contents leaking and bacteria rupture. Gazvoda et al. gave a clear explanation of transmembrane current‐incudes electroporation, that was positive charges could generate an endogenous electric field together with bacterial membrane, resulting in the lipids distraction and loss of electron.^[^ ^172^ ^]^ This triggered the membrane damage and transmembrane potential disruption, and then destroy the normal cell morphology leading to the bacterial death. The SEM images clearly reflected the damage of the S. epidermidis cell envelope (Figure 10f).

Meanwhile, ROS generated by piezoelectric ceramic has proved excellent bactericidal property through inducing peroxidation of polyunsaturated phospholipid in lipid membrane and altering the selective permeability. This will lead to the intracellular components leakage and kill the bacteria ultimately.^[^ ^173^, ^174^ ^]^ In 2023, Liu et al. evaluated the antibacterial capability of BTO doped piezoelectric hydrogel through the quantitative analysis of bacterial protein leakage. They found that the mount of bacterial protein leakage increased together with the US irradiation time prolonging.^[^ ^139^ ^]^ This was further confirmed by fluorescent images of bacteria live/dead staining assay quantitative results that 98.5% and 97.4% of dead *E. coli* and *S. aureus* after 10 min US explosion (Figure 10g,i). Furthermore, Geng et al. compared the micromorphology of bacteria cultivated on their piezoelectric materials with untreated ones to confirm the cell membranes destruction through various detection methods.^[^ ^106^ ^]^ The field emission scanning electron microscopy (FE‐SEM) revealed the cell membrane shrinkage and deformation, while TEM detected internal cytoplasm leakage and decrease in intracellular density. Additionally, they employed AFM on DRB and observed a significant increase in roughness and reduction of overall height, also indicating the alteration of membrane structure and cytoplasmic leakage (Figure 10h).

### 4.3 Energy Metabolism Interruption

**Figure 11:**
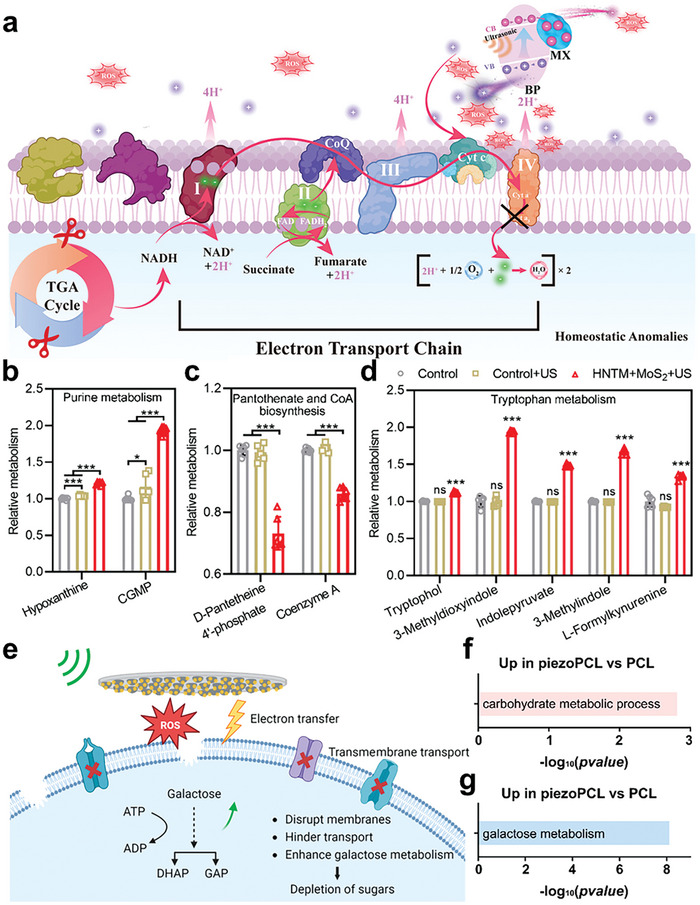
a) Schematic diagram of P‐BM antibacterial effect via electron transport chain. Reproduced with permission.^[^ ^106^ ^]^ Copyright 2024, John Wiley and Sons. The relative b) purine metabolism, c) pantothenate and CoA biosynthesis and d) tryptophan metabolism comparison in different group. Reproduced with permission.^[^ ^104^ ^]^ Copyright 2022, American Chemical Society. e) Schematic illustration of piezoPCL‐induced bacterial inactivation mechanisms. f) Gene ontology (GO) enrichment analysis of upregulated carbohydrate metabolic pathways and g) KEGG enrichment analysis of enhanced galactose metabolism processes in peizoPCL versus PCL. Reproduced with permission.^[^ ^7^ ^]^ Copyright 2023, American Chemical Society.

Previous studies have widely demonstrated that polarized piezoelectric materials can effectively eliminate bacteria by disrupting metabolism and electron transport chain (ETC). In 2024, Geng et al. thoroughly investigated the inhibitory effects of their piezoelectric heterojunction materials on bacterial metabolism.^[^ ^106^ ^]^ Their findings revealed a significant impact on the activity of complex enzymes IV, disrupting the flow of electrons, impeding ATP synthesis, and ultimately leading to a substantial suppression of bacterial ATP production. They also contrasted the Genomes (KEGG) enrichment analysis on the transcriptomes of drug‐resistant *E. coli* cultured on piezoelectric materials and under normal growth conditions. The results demonstrated that carbon metabolism, glycolysis/gluconeogenesis, and citrate cycle (TCA cycle) showed the most significant difference, indicating that bacterial energy supply was affected. The analysis of differentially expressed genes revealed noteworthy patterns: genes associated with tryptophan metabolism (trpA, trpB, trpC, trpD, and trpE) and lipopolysaccharide (LPS) modifications (waaH, ugd, and eptA) showed upregulation. Conversely, genes linked to membrane integrity (osmB, ynfT, and yaiY), and bacterial growth (ygeX) were downregulated. (**Figure** **11**a) This indicated that the induced oxidative stress resulted in significant disruptions in metabolic pathways and intracellular stress among affected microbes.

Similar mechanisms were also mentioned by Feng et al. in their metabolomics study of piezo‐augmented sonosensitizer for MRSA treatment.^[^ ^104^ ^]^ The KEGG enrichment analysis of piezoelectric and control group showed significant statistical differences. Specifically, there was a marked increase in tryptophan metabolism and purine metabolism, alongside a notable suppression of pantothenate and CoA biosynthesis. (Figure 11b–d) The excessive metabolite of tryptophan revealed the capacity of piezoelectric material to generate abundant ROS and exhibit stronger oxidative stress.^[^ ^175^ ^]^ The over‐enhancement of purine metabolism suggested accelerated nucleotide turnover and a higher incidence of DNA damage. Furthermore, the decreased pantothenate and CoA biosynthesis pose a threat for membrane biogenesis and TCA cycle.

Other than TCA cycles mentioned above, Xu et al. studied the transmembrane substance transport process of bacterial membranes and discovered that the oxidative stress and surface electron enrichment stimulate galactose metabolism.^[^ ^7^ ^]^ Galactose plays a vital role in human metabolism, serving as an essential component for energy supply and the process of galactosylation in complex molecules. Galactoses are actively transported across the membrane by the Na^+^/glucose cotransporter or symporter sodium/glucose cotransporter 1 (SGLT1).^[^ ^176^ ^]^ The inhibition of transmembrane substance transport decreases the production of glycolytic intermediates: dihydroxyacetone phosphate (DHAP) and glyceraldehyde 3‐phosphate (GAP), which are essential in the generation of ATP.^[^ ^177^ ^]^ In their work, the modified piezoPCL they synthesized posed great threat for bacterial membrane stability and function, as well as downgraded the transmembrane transporter‐related genes. (Figure 11e–g) This implies while the demand for energy supply for membrane repairment increased, carbohydrates metabolism was instead cut down on the other hand. This ultimately results in an increased consumption of sugar and starvation, leading to the death of stressed bacteria.

### 4.4 Macrophage Polarization Regulation

Biomaterial mediated immunotherapy is another promising approach for infection control.^[^ ^178^, ^179^ ^]^ Given their pivotal role of antimicrobial effector cells within the innate immune system, regulating the activation of macrophages is a crucial endeavor. When exposed to pathogens, macrophages typically exhibit M1 phenotypes upregulate pro‐inflammatory factors to combat infection through phagocytosis and antigen dependent facets.^[^ ^180^ ^]^ Unluckily, the existence of dense biofilm formation results in frustrated phagocytosis of macrophage and even changes the polarization from M1 towards the anti‐inflammatory phenotype of M2. Such immune evasion mechanisms have been widely recognized in *S. aureus*.^[^ ^181^, ^182^ ^]^ To address this issue, researchers have focused on activating proinflammatory immunity via ROS production from catalytic nanoparticles, after realizing that ROS can induce the expression of various pro‐inflammatory cytokines in immune cells.^[^ ^183^, ^184^ ^]^

**Figure 12:**
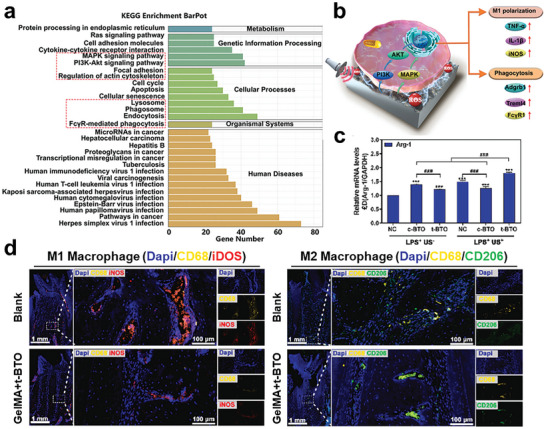
a) KEGG enrichment of piezoTi with and without US; b) Schematic diagram of piezoelectric‐induced macrophage polarization molecular signaling. Reproduced with permission.^[^ ^125^ ^]^ Copyright 2023, John Wiley and Sons. c) Expression of Arg‐1 in L‐RAW264.7 cells following different treatments; d) Fluorescence images of M1 and M2 macrophage markers in the BTO and blank groups. Reproduced with permission.^[^ ^187^ ^]^ Copyright 2024, Elsevier.

Investigating the effects of ROS produced by piezoelectric materials on the attached macrophages, Li et al. found that the macrophages tend to exhibit a larger size and grow multiple extended pseudopodia, indicating a transformation in morphology towards the proinflammatory M1 status.^[^ ^125^ ^]^ The upregulation of typical M1 markers (TNF‐α, IL‐1β, IL‐6, and iNOS) in gene expression results further supports this conclusion (**Figure** **12**a,b). In detail, after 12 h of culture under US stimulation, there was a notable upsurge in the expression of M1 makers, with the proportion of positive cells potentially reaching at least 40%. They also found that polarized piezo‐surface was able to enhance the phagocytic ability of attached macrophages through the enrichment of PI3K‐AKT and MAPK signaling pathways and FcγR‐mediated innate immune phagocytosis. The decreased expression levels of Arhgap12 and Arhgap25, the inhibitors of phagocytosis by regulating the actin cytoskeleton, further substantiates this observation.^[^ ^185^ ^]^ To confirm the role of piezoelectric materials in inducing macrophages to enhance phagocytosis, the flow cytometer was utilized and the high phagocytosis rate was indicated of piezo‐surface. In macrophage‐*S. aureus* biofilm coculture experiment, the piezo surface cultivated only 1% or so *S. aureus* colonies with US assistance, while other control groups exhibited almost no antibiofilm ability.

However, maintaining immune balance demands careful attention. While enhancing M1 polarization enables macrophages to express pro‐inflammatory factors more intensely for efficient antibacterial responses, sustained macrophage‐mediated inflammation inhibits the formation and maturation of new blood vessels and slows tissue healing, which run counter to efficient bacterial clearance and tissue regeneration purpose.^[^ ^186^ ^]^ Thus, the M2 polarization modulation of macrophages is often utilized for anti‐inflammatory and pro‐regenerative niche after antibacterial procedure. Liu et al. demonstrated that their BTO nanoparticles with US radiation could significantly ungraded expression of arginase 1 (Arg‐1), which is a specific biomarker of M2 macrophage (Figure 12c).^[^ ^187^ ^]^ Further immunofluorescence staining of this BTO composite piezoelectric hydrogel in Figure 12d revealed decreased M1 and increased M2 macrophages in the periodontal defect model, confirming a balanced macrophage phenotype and pro‐regenerative immune microenvironment establishment.

In terms of the current knowledge of antibacterial therapies, the enhancement of phagocytosis through M1 polarization is effective in bacterial elimination, whereas M2 polarization is responsible for anti‐inflammatory and immune balance in the subsequent stages of therapy. As the research on the regulation of macrophages by piezoelectric materials is still in its preliminary stage, more effort is required for a clear cognition of the specific interaction mechanism between piezoelectric materials and macrophages.

## 5 Antibacterial Applications in Different Tissue Repair

Bioelectric effect is an important phenomenon in biological microenvironment. It is not only a sharp tool in antibacterial processes, but also a decisive factor in tissue regeneration. As an exogenous stimulation responsive material, piezoelectric materials offer a promising solution for infectious tissue replacement. These materials exhibit potent antimicrobial effects; however, careful design is essential to minimize any potential cytotoxicity to surrounding healthy tissues. Recent advancements in surface modification and structural optimization have led to piezoelectric biomaterial with improved biocompatibility, promoting cellular attachment, proliferation, and differentiation without inducing significant toxicity. The charged surface as well as the ROS generation equip piezoelectric materials with a remarkable advantage in facilitating both antimicrobial action and tissue regeneration, which is a well‐documented phenomenon. As hard tissue alternatives such as bone and dental, the piezoelectric grafts require certain mechanical strength, durability, osteogenesis, and osteointegration abilities.^[^ ^188^ ^]^ When it comes to soft tissue engineering, the attention will turn to cell migration, inflammatory factor release, collagen deposition and so on. At present, the piezoelectric materials used for soft tissue healing mostly focus on skin regeneration, and a few of studies explored the application in other aspects such as intestines and cartilage.^[^ ^147^, ^166^ ^]^

### 5.1 Bone Tissue Regeneration

Bacterial invasion may occur through any processes of bone defect treatment including open wounds, improper operation, and the migration of bacteria from infectious diseases in other parts of the body.^[^ ^189^ ^]^ Despite the advancements in aseptic techniques and minimally invasive surgery, the incidence of orthopedic infection range from 0.1% to 30%, with corresponding costs per patient ranging from $17 000 to $150 000 according to the 2018 International Consensus Meeting on Musculoskeletal Infection.^[^ ^190^ ^]^ Even worse, there is an increasing possibility of infection recurrence as time passes after surgical implantation.^[^ ^191^ ^]^ Osteomyelitis is a severe bone infectious syndromes infection with rising incidence recent years, especially in the elderly and individuals with diabetes.^[^ ^192^ ^]^ Staphylococcus are responsible for 75% of osteomyelitis cases, more than two‐thirds of which are attributed to MRSA due to the despairing antibiotic abuse.^[^ ^193^ ^]^ However, the application of piezoelectric materials presents a promising avenue for antibacterial therapy through the bioelectric pathway rather than relying on antibiotics.

**Figure 13:**
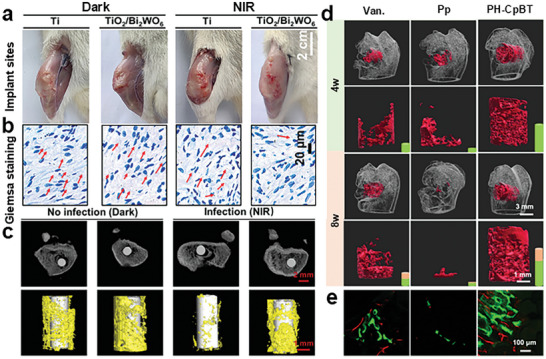
a) Photographs of the implant sites; b) Giemsa staining of bacteria distribution around the implants; c) Micro‐CT images of bone repair around different implants.^[^ ^154^ ^]^ Copyright 2024, John Wiley and Sons. d) The representative images of new bone ingrowth in the scaffolds after 4 weeks (green) and 8 weeks (pink) implantation through Micro‐CT and fluorescent staining; e) New bone formation labeled with fluorescence (green: calcitonin, red: alizarin red). Reproduced with permission.^[^ ^152^ ^]^ Copyright 2024, Springer Nature.

To graft bone implant with antibacterial ability, piezoceramics are always utilized to modified bioinert implant such as Ti alloy and PEKK. For example, Fan et al. decorated TiO2 nanowires on Bi2WO6 nanocrystals to construct a piezo‐heterojunction and incorporate in Ti implant.^[^ ^154^ ^]^ It is confirmed that this piezoelectric heterojunction leverages photodynamic and photothermal therapies to generate ROS for bactericidal effects, while the internal electric field promotes osteogenesis in stem cells, aiding bone integration. A rat femoral prosthesis infection model was developed to evaluate the antibacterial and osteogenic effects of the piezoelectric heterojunction implants compared to pure titanium, with and without near‐infrared light exposure. Under 7 d of NIR irradiation, only the TiO_2_/Bi_2_WO_6_+ NIR group demonstrated the most effective antibacterial treatment, with no signs of inflammatory exudation or pus at the implantation sites (**Figure** **13**a). The Giemsa staining of implant infection tissues also proved this conclusion in Figure 13b. The bone formation capacity was demonstrated by quantitatively analyzing the microcomputed tomography (micro‐CT) (Figure 13c). The TiO_2_/Bi_2_WO_6_ implants showed superior repair effects compared to pure titanium implants in the treatment of infectious bone defects, with performance nearly identical to that under non‐infected conditions. Meanwhile, Huang et al. designed a nanoreactor bridging Cu and BTO with polydopamine.^[^ ^152^ ^]^ Harnessing this innovation, they integrated the nanoreactor into a polyetherketoneketone (PEKK) scaffold, exhibiting a remarkable enhancement in antibacterial efficacy. The in vivo studies revealed the scaffold's capacity to precisely induce angiogenesis and osteogenesis as well. With the nanoreactor assistance, new bone effectively filled the scaffold pores and exhibited outstanding bone formation in comparison to vancomycin. The green and red labeled area representing the new bone deposition in 4 and 8 weeks separately, which identified the rapid bone deposition rate (Figure 13d,e).

### 5.2 Dental Tissue Engineering

**Figure 14:**
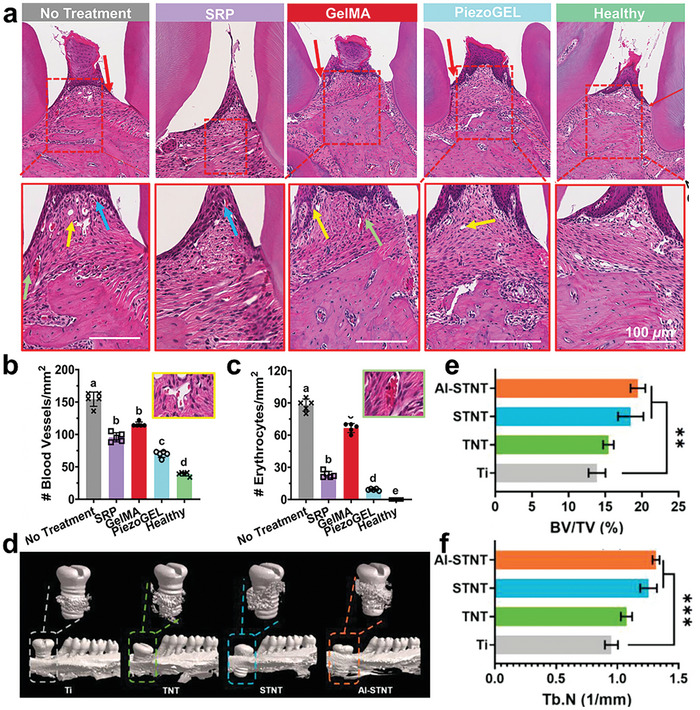
a) hematoxylin and eosin (H&E) stain and magnification inset of periodontal tissue sections; b) Quantified blood vessels and c) erythrocytes of tissue in the box. Reproduced with permission.^[^ ^151^ ^]^ Copyright 2023, American Chemical Society. d) 2D and 3D reconstructions of dental implants and surrounding bone tissues via Micro‐CT; e) Trabecular bone volume fraction (BV/TV) and f) trabecular number (Tb. N) of the surrounding bone tissue. Reproduced with permission.^[^ ^100^ ^]^ Copyright 2024, John Wiley and Sons.

If not addressed in a timely manner, the inflammation caused by microbial infection and tooth trauma can lead to dental damage and ultimately the loss of periodontal tissues or the need for endodontic treatment.^[^ ^188^ ^]^ The chronic bacterial infectious disease, periodontitis, is highly prevalent and affects more than 90% of the global population.^[^ ^194^ ^]^ The high prevalence of periodontitis can be attributed to the presence of subgingival plaque biofilm, primarily consisting of Gram‐negative bacteria.^[^ ^84^ ^]^ Given that dentin can generate piezoelectric potential in response to mechanical stimulation, it has sparked considerable interest in the advancement of mechano‐responsive, piezoelectric biomaterial‐based scaffolds with the purpose of repairing and regenerating dental tissues.^[^ ^195^, ^196^ ^]^ For instance, Roldan et al. combined gelatin methacryloyl (GelMA) with BTO fillers and developed an injectable piezoelectric hydrogel tailored for the treatment of periodontal disease.^[^ ^151^ ^]^ This piezoGEL showed effective periodontal inflammation reduction. In histological sections, there were less than 50% of the blood vessels and nearly one in six of erythrocytes comparing to GelMA and no treatment groups while perform the most similar to healthy groups, as redundant blood vessels supply nutrients and oxygen for infectious tissue to maintain the inflammation process (**Figure** **14**a–c).

Dental implants replace missing teeth by attaching them directly to the alveolar bone. Without the attachment of connective tissue, this direct connection makes them prone to bacterial plaque accumulation.^[^ ^197^ ^]^ Persistent plaque buildup leads to inflammation in the surrounding soft tissues and eventual deterioration of the alveolar bone.^[^ ^198^ ^]^ Targeting this issue, Pan et al. designed a Ti dental implant with Al doped SrTiO_3_/TiO_2_ nanotubes decoration, trying to implement an effective solution for plaque accumulation around implant.^[^ ^100^ ^]^ The application of SrTiO_3_ coating endowed dental implants with osteogenic properties, fostering the development of robust osseointegration between the implant surface and the alveolar bone. They implanted the dental implant into the alveolar bone in front of the maxillary first molar of the rats and raised it for 4 weeks. 3D micro‐CT images had emerged of maxilla and bone formation around, visualizing the new bone formation and bone trabeculae. The incorporation of SrTiO_3_ and Al‐doped SrTiO_3_ led to a notable increase in bone volume fraction, reaching 133% and 140% respectively compared to pure Ti (Figure 14f). Additionally, these coatings exhibited a higher degree of bone trabeculae amount, suggesting a superior osseointegration strength (Figure 14d,e).

### 5.3 Wound Healing

Skin is not only the largest organ of human body but also acts as the body's primary bacterial defense system.^[^ ^199^ ^]^ When damaged, its protective and defensive functions are compromised, thereby posing a significant risk to individuals' health and overall safety. Wound defect repair is a lengthy process, during which the disordered deposition of collagen fibers and abnormal remodeling of the extracellular matrix (ECM) occur, delaying the healing process.^[^ ^155^ ^]^ Infection is considered the major factor in delaying the wound healing because of the opportunistic colonization by bacteria, i.e., Staphylococci, Pseudomonas, and Acinetobacter strains, etc.^[^ ^200^ ^]^ Consequently, the discharge of substances from the wound bed, such as endotoxins, can stimulate inflammatory reactions, ultimately resulting in insufficient wound healing, invasive infection, and potential sepsis.^[^ ^201^ ^]^ the typical treatment for infected wounds involves both eliminating pathogens and promoting tissue regeneration.

**Figure 15:**
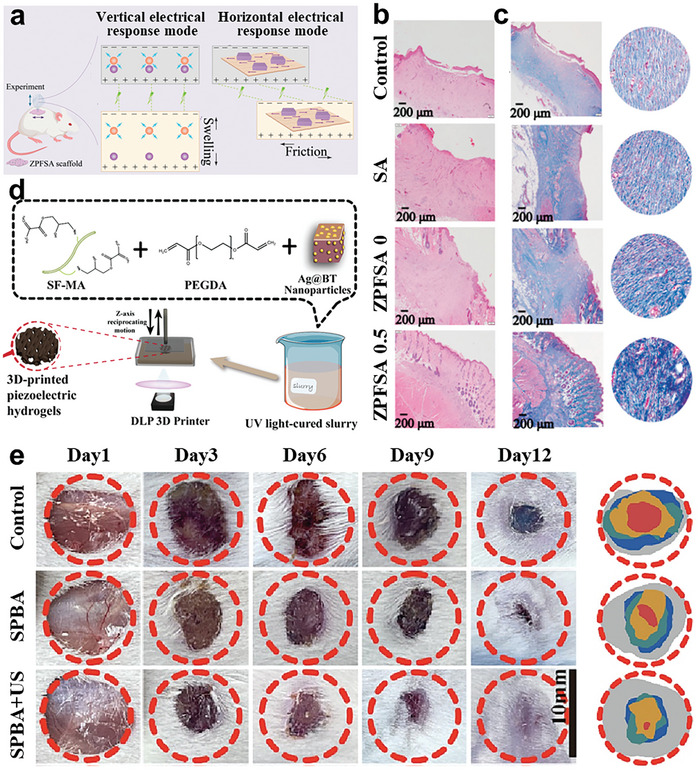
a) H&E staining and b) Masson staining of wound tissue treated by different samples on 14 d. Reproduced with permission.^[^ ^155^ ^]^ Copyright 2022, American Chemical Society. c) Scheme illustration of the preparation process of piezoelectric hydrogel dressing; d) Wound healing images of hydrogel dressing compared to control group. Reproduced with permission.^[^ ^148^ ^]^ Copyright 2024, Elsevier.

Piezoelectric hydrogel patches are the most used biomaterials for wound healing for its high moisture content and their ability to mimic ECM. The piezoelectricity of hydrogel derives from the composition of piezoelectric materials in the shape of particle or fiber. Liang and colleagues developed a piezoelectric hydrogel scaffold, which is made of polyvinylidene fluoride (PVDF) and sodium alginate (SA) with ZnO modification (**Figure** **15**a).^[^ ^155^ ^]^ The piezoelectric scaffold exhibits dual piezoelectric responses stimulating bioelectric effects, which help to promote wound healing and prevent scar formation.^[^ ^202^ ^]^ As shown in Figure 15b,c, the piezoelectric group exhibits advantages in mitigating inflammatory responses, facilitating angiogenesis and re‐epithelialization. Additionally, there was an enhanced collagen deposition and a well‐organized arrangement of fiber networks at the wound site, fostering tissue remodeling. In 2024, Chen et al. mixed Ag‐BTO heterojunctions with silk fibroin/poly (ethylene glycol) diacrylate dual‐network hydrogel and fabricated it using digital light processing, which scheme is shown in Figure 15d.^[^ ^148^ ^]^ This piezoelectric hydrogel dressing features pores with suitable dimensions for tissue fluid exchange and can generate ROS under ultrasound stimulation to rapidly kill bacteria. In vivo experiments showed complete healing of a 10 mm bacterial‐infected wound within 12 d (Figure 15e).

## 6 Summary and Perspective

Pathogenic bacteria pose significant threats to public health and are encountered by humankind on a severe level. The existence of biofilm prevents the permeate of antibiotic and decrease the bacteria killing effect to a large extent, which leads to the abuse of antibiotic and the occurrence of multiresistant microbial. Piezoelectric materials exhibit large potential in bacterial processes owing to its noninvasive stimulation ability. Piezoelectricity and piezocatalysis endow piezoelectric materials with advancement in bacterial eradication and tissue repair by means of electromechanical conversion and catalyzing ROS generation. This review delivers the recent signs of progress in various piezoelectric implantations for antibacterial purposes from a different perspective. Beside the structural demonstrations of common piezoceramic and piezopolymer, detailed illustrations are provided on the principles of piezoelectric response and piezoelectric catalysis to enhance readers’ understanding of piezoelectric materials operational mechanism. Moreover, this review summarizes the bacteria killing pathways of piezoelectric materials from the biofilm damage in the microenvironment to the bacterial respiratory chain interruption inside mitochondria. In addition, some recent progress in the configuration of piezoelectric implantation is discussed, with emphasis on the design strategies to enhance antibacterial effect as well as the combination of piezoelectric materials with implant substrate. Finally, the latest biomedical applications of piezoelectric biomaterial in terms of bone scaffolds, dental implants wound healing patches are concluded. As an emerging research interest, many research and conclusions have been done focusing on the antibacterial applications of piezoelectric materials. Compared with the most recent review,^[^ ^203^ ^]^ this work investigates infection therapy from the perspective of both the antibacterial mechanisms and tissue regeneration effect of piezoelectric biomaterials. Inspired by tissue engineering, this review does not only focus on the design of piezoelectric materials to perform antibacterial functions but also aim to combine these materials with biomaterial scaffolds to enhance tissue repair during the antibacterial process.

Piezoelectric biomaterial integrates electromechanical coupling, non‐invasive response, ROS generation ability, making them prospective functional biomedical materials in antibacterial therapy. Despite the initial progress that has been made, there remain numerous challenges requiring further deliberation and effort. Although the present piezoelectric biomaterial is typically composed of multiple components and exhibits a variety of combinations, there is a lack of overall consideration regarding the interrelationships among these components. Common strategies of hard tissue infectious regeneration systems are based on Ti alloy or HA substance with additional piezoelectric coating or blending, whereas soft tissue repair typically incorporates a combination of piezoelectric dispersed phases and organic continuous phases like PLLA, PCL, and hydrogel. However, these materials collocations are merely selected due to the consideration of mechanical properties and biocompatibility, making it challenging to achieve a “1 + 1 > 2” synergistic effect. Meanwhile, the synthetic production techniques are supposed to keep pace with advancements in piezoelectric biomaterial design for the purpose of further clinical treatment possibility. Furthermore, the current biomedical applications of piezoelectric materials are commonly ultrasound imaging, piezoelectric sensors or electrical stimulation devices. A path to the clinical therapeutic use of piezoelectric biomaterial in antimicrobial medical products remains challenging, requiring rigorous clinical trials, premarket evaluations for industrialization and commercialization, and the establishment of a robust regulatory framework—all of which demand considerable effort and research.^[^ ^204^ ^]^ We believe that the increasing in vitro and in vivo exploration of piezoelectric biomaterial as biomedical devices will accelerate their development and unlock promising potential. Moreover, except for antimicrobial, the applications of piezoelectric materials with other functions like antitumor and flame‐retardant are also investigated. Exploring the interconnections among antimicrobial, antitumor, flame‐retardant properties, and other therapeutic avenues offers valuable insights and warrants further in‐depth research. Although, antibacterial application of piezoelectric materials is still in its early stages, it is predictable that a significant progress will be made to understand the deep relationship of piezoelectricity and antimicrobial, which may further increase treatment effectiveness and extend the application feasibility.

## Conflict of Interest

The authors declare no conflict of interest.
